# Hole Transport Materials for Tin-Based Perovskite Solar Cells: Properties, Progress, Prospects

**DOI:** 10.3390/molecules28093787

**Published:** 2023-04-28

**Authors:** Xinyao Chen, Jin Cheng, Linfeng He, Longjiang Zhao, Chunqian Zhang, Aiying Pang, Junming Li

**Affiliations:** 1 School of Instrument Science and Opto Electronics Engineering, Beijing Information Science and Technology University, Beijing 100101, China; chenxinyao0206@163.com; 2 Beijing Key Laboratory for Sensor, School of Instrument Science and Opto-Electronics Engineering, Beijing Information Science and Technology University, Beijing 100101, Chinazhangcq@bistu.edu.cn (C.Z.);; 3 College of Engineering, Qufu Normal University, Rizhao 276826, China; 4 Jiangsu Engineering Laboratory for Environmental Functional Materials, School of Chemistry and Chemical Engineering, Huaiyin Normal University, Huaian 223300, China

**Keywords:** perovskite solar cell, tin-based, hole transport materials, energy level matching

## Abstract

The power conversion efficiency of modern perovskite solar cells has surpassed that of commercial photovoltaic technology, showing great potential for commercial applications. However, the current high-performance perovskite solar cells all contain toxic lead elements, blocking their progress toward industrialization. Lead-free tin-based perovskite solar cells have attracted tremendous research interest, and more than 14% power conversion efficiency has been achieved. In tin-based perovskite, Sn^2+^ is easily oxidized to Sn^4+^ in air. During this process, two additional electrons are introduced to form a heavy p-type doping perovskite layer, necessitating the production of hole transport materials different from that of lead-based perovskite devices or organic solar cells. In this review, for the first time, we summarize the hole transport materials used in the development of tin-based perovskite solar cells, describe the impact of different hole transport materials on the performance of tin-based perovskite solar cell devices, and summarize the recent progress of hole transport materials. Lastly, the development direction of lead-free tin-based perovskite devices in terms of hole transport materials is discussed based on their current development status. This comprehensive review contributes to the development of efficient, stable, and environmentally friendly tin-based perovskite devices and provides guidance for the hole transport layer material design.

## 1. Introduction

In the face of the global energy crisis and environmental pollution, solar energy, as a renewable and clean energy source, is undoubtedly one of the most effective solutions. Moreover, by employing the photovoltaic effect, which converts solar energy into electricity, the solar energy approach presents as the most promising solution. At present, the main types of solar cells include inorganic semiconductor solar cells, organic semiconductor solar cells and perovskite solar cells. Perovskite solar cells, which use halide perovskite as the light-absorbing material, have received ample attention in the past decade. In 2009, Kojima et al. first introduced the perovskite structure of methylammonium lead iodide (MAPbI_3_) and methylammonium lead bromide (MAPbBr_3_) into dye-sensitized solar cells, achieving a photovoltaic conversion efficiency (PCE) of 3.8% [1]. In 2012, Nam-Gyu Park’s group and Michael Grätzel’s group collaborated to prepare the first all-solid-state perovskite solar cell with a cell efficiency of 9.7%; instead of liquid electrolyte, they utilized the solid-state hole-transport material 2,2′,7,7′-tetrakis(N,N-di-p-methoxyphenyl-amine)9,9′-spirobifluorene (Spiro-OMeTAD) [2]. In the same year, an efficiency of 10.9% was achieved by Henry J. Snaith’s group; instead of TiO_2_, they utilized Al_2_O_3_ as the hole transport material with a mesoscopic structure [3]. After breaking through the 10% efficiency bottleneck, perovskite solar cells have been rapidly improved upon both in materials design and device fabrication technique. So far, the certified energy conversion efficiency (PCE) of perovskite solar cells is 25.7% [4], which already surpasses that of CdTe (22.1%), polycrystalline silicon (23.3%), and copper indium gallium selenide (23.4%) solar cells, making it one of the most intensive research topics in the field of solar cells [3,5,6,7,8,9,10,11,12,13,14,15]. Although perovskite solar cells have undergone extremely rapid development in recent years, there are still, however, many problems to be solved to realize the commercialization of perovskite solar cells. One of the most pressing issues is that the current high-performance perovskite solar cells all contain lead. Since the thermal and chemical stability of lead-based perovskite materials are relatively poor, most or all of the lead would be decomposed into the environment [16]. In particular, we found that these leaked lead ions enter the soil in a free state and they were absorbed by plants, and finally enter the food chain. Compared to the lead in the natural environment, the absorption capacity of plants for lead in perovskite materials is 10 times higher [17]. 

To replace the Pb ions with equivalent elements, the main B-metal elements that satisfy both the tolerance factor *T* and octahedral factor *μ* are tin (Sn), calcium (Ca), strontium (Sr), barium (Ba), bismuth (Bi) and tellurium (Te). In addition, it was found that when replacing Pb, the replaced element needs to hybridize effectively with the outer orbitals of the halogen group elements, to form the corresponding conduction and valence bands. Among these substitution materials, Sn has a similar shell electron configuration to Pb, a smaller ionic radius (Sn: 0.110 nm, Pb: 0.119 nm), a lower atomic mass and a higher 5s orbital energy level. Thus, the Sn-based perovskite has a similar energy level structure to Pb-based perovskite, with a band gap of about 1.30 eV, which is closer to the ideal band gap for solar cells than that of Pb-based perovskite. Meanwhile, tin-based perovskite materials have excellent photovoltaic properties such as high carrier mobility, high absorption coefficient in the visible region, and long exciton lifetime. The precise origin of these excellent opto-electronic properties is still largely unclear, and the fundamental understanding of charge migration processes will be important for further device optimization. One important point is that the migration properties of light-produced holes are determined by corresponding perovskite structures from the first-principles (ab initio) computations [18,19,20]. Thus, tin is one of the most likely alternatives to the lead-based perovskites [21,22]. However, the Sn^2+^ element is easily oxidized to Sn^4+^ under the presence of oxygen or water. Density function theory simulations suggest that the oxygen or water, by the formation of H-I or Sn-O bonds, would affect the Sn-I bonds in the tin perovskite, which is less significant in the case of lead perovskite [23]. In the PbX_2_ for the lead perovskite, two electrons were donated from the Pb^2+^ cation 6p orbital to the p orbitals of two X^−^ anions [24].

In 2014, Kanatzidis’s group and Snaith’s group almost simultaneously reported the first tin-based perovskite solar cells, with MASnBr_3_ (MA=CH_3_NH_3_) or MASnI_3_ as the active layer. The power conversion efficiency was around 6% [25,26]. However, the device reproducibility of these tin-based perovskite solar cells is low. Recently, the strategy of 2D-3D (quasi-2D) perovskite materials, which are 3D perovskite materials, doped with a small amount of 2D perovskite materials, has showed great potential for the development of efficient and stable tin-based perovskite cells. In quasi-2D perovskite solar cells, the amine molecules in 2D perovskite can coordinate with the organic cations in 3D perovskite, so that the 2D perovskite becomes implanted at the 3D perovskite crystal plane surface. It can effectively inhibit the non-radiative recombination at the grain boundaries and prevent the diffusion of water and oxygen into the internal 3D perovskite. In 2017, Liao et al. replaced the A-site ions FA^+^ in the 3D perovskite material FASnI_3_ (FA=CH(NH_2_)_2_) with large-size phenylethylammonium ions (PEA^+^) for the first time; the original halogen octahedra expanding in the 3D direction to be isolated by these large-size ions, resulting in an increase in open-circuit voltage from 0.26 V undoped to 0.59 V, and the efficiency was increased to 6% [27]. Recently, Jiang et al. obtained a certified photovoltaic conversion efficiency of 14.6% by designing a new synthetic route that allows the fabrication of high-quality tin-perovskite films with fewer pinholes, more uniform orientation, and substantially increased carrier diffusion length [28]. Currently, the research focus has shifted from regular (n-i-p) to inverted (p-i-n) due to problems in the regular structure such as the diffusion length of holes being much shorter than that of electrons, the necessary chemical doping of hole transporting materials and the oxygen vacancies on the surface of titanium dioxide that accelerate Sn^2+^ oxidation. Meanwhile, the low open-circuit voltage is the main reason for the low performance of tin-based perovskite cells, thus now the main focus of research is on how to minimize the voltage loss, through the reduced defect density and the energy level alignment at the perovskite/charge transport layer interface.

Recently, several reviews on tin-based perovskite material design and fabrication technique have been published. However, there were few reviews devoted to the hole transport layer. The hole transport layer, which is an important link in the structure of a perovskite solar cell for transporting holes and blocking electrons, has been well studied and selected for good performance in lead-based perovskite solar cells. Sn-X of Sn-based perovskites has a stronger s-p antibonding orbital than Pb-X, giving it a shallower conduction band and valence band position. In addition, the use of metal dopants should be avoided due to the instability of tin-based materials. This leads to the fact that the design experience of previous hole transport materials in lead-based perovskite solar cells and organic solar cells is not applicable to the tin-based perovskite devices, so it is necessary to summarize the study of hole transport materials in tin-based perovskite solar cells. In this work, we first summarize the current progress of tin-based perovskite solar cells using different hole transport materials, then describe the impact of different hole transport materials on the performance of tin-based devices, and finally discuss the direction of development of lead-free tin-based perovskite devices in terms of hole transport materials. This comprehensive review contributes to the environmentally friendly tin-based perovskite solar cell device structures and provides guidance for the realization of efficient and stable non-lead perovskite solar cells through material design.

## 2. Classical Hole Transport Layer: Spiro-OMeTAD

**Figure 1:**
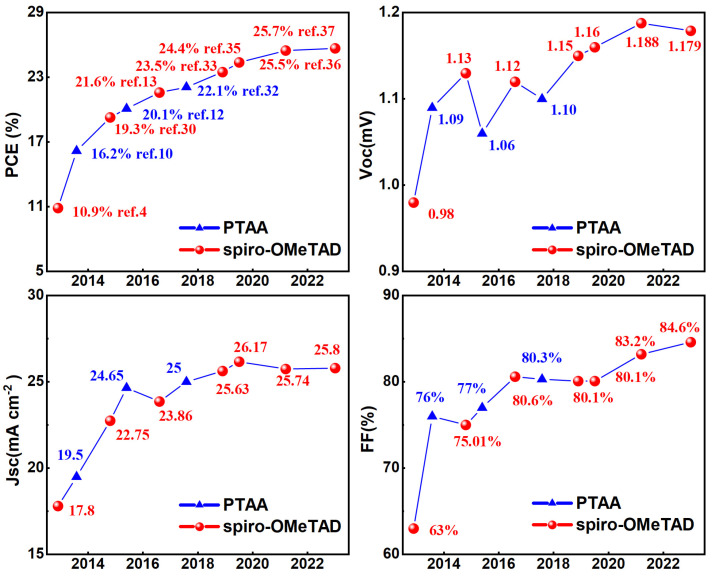
Performance of perovskite solar cells with recorded efficiency over the last decade [2,3,4,10,12,13,29,30,31,32,33,34,35,36,37].

The initial introduction of tin-based perovskite solar cells has benefitted from the successful experiences gathered from lead-based perovskite solar cells. The most classic hole transport material in lead-based perovskite solar cells is Spiro-OMeTAD. In 2012, Spiro-OMeTAD was introduced for the first time to prepare the all-solid-state perovskite solar cell as the hole transport material [2]. Today, the highest efficiency of perovskite solar cells is 25.7%, also using Spiro-OMeTAD as the hole transport layer. Figure 1 summarizes the hole transport materials used for the highest efficiency of perovskite solar cells [2,3,4,29,30,31,32,33,34,35,36,37]. To obtain Spiro-OMeTAD films with high hole mobility and conductivity, typically three preparation processes are used: (1) addition of P-type dopants lithium bistrifluoromethanesulfonimide (Li-TFSI) and 4-tert-butylpyridine (tBP): Li-TFSI regulate the reaction of O_2_ with Spiro-OMeTAD under illumination, and provide TFSI^−^ stable radical cations along with lithium oxides. Such an oxidation process relies on very slow oxygen entry in air and diffusion through Spiro-OMeTAD:Li-TFSI, as follows [26]:

(1) $$\mathrm{Spiro}-\mathrm{OMeTAD}+O_{2}↔{\mathrm{Spiro}-\mathrm{OMeTAD}}^{.+}O_{2}^{.-}$$

(2) $${\mathrm{Spiro}-\mathrm{OMeTAD}}^{.+}O_{2}^{.-}+\mathrm{Li}-\mathrm{TFSI}\to {\mathrm{Spiro}-\mathrm{OMeTAD}}^{.+}{\mathrm{TFSI}}^{-}+{\mathrm{Li}}_{x}O_{y}$$

(2) Addition of the strong oxidation dopant tris(2-(1H-pyrazol-1-yl)-4-tert-butylpyridine) cobalt (III) tris-(bis(tri-fluoromethane) sulfonimide) (FK209) to the Spiro-OMeTAD:LiTFSI:tBP solution, to replace the slow oxidation process; (3) exposure to UV light excitation for Spiro-OMeTAD:LiTFSI solution with CO_2_ gas bubbling treatment, in which CO_2_ gains electrons from photoexcited Spiro-OMeTAD to achieve p-doping [38,39], as follows:

(3) $$4{\mathrm{Li}}^{+}+3{\mathrm{CO}}_{2}+4e^{-}\to 2Li_{2}CO_{3}+C$$

### 2.1. Limitations of Pure Spiro-OMeTAD

Before copying the success of lead-based perovskite solar cells with Spiro-OMeTAD as the hole transport layer, the tin-based perovskite layer faces an inevitable problem: when exposed to acetonitrile solution of tBP:Li-TFSI, the hygroscopic properties of the lithium salt therein accelerate the oxidation of Sn^2+^ to Sn^4+^; thus, even with rapid cobalt doping processing, the degradation of the tin-based perovskite film cannot be avoided. When the undoped Spiro-OMeTAD was used as the hole transport layer, Wang et al., Kumar et al. and Ke et al. found that although from the perspective of tin-based perovskite films, undoped Spiro-OMeTAD fundamentally solved the problem of additive-induced film instability, respectively. However, in terms of overall device performance, undoped Spiro-OMeTAD could not achieve high conductivity without doping or oxidation, which limits the charge transport in the hole transport layer and thus limits the performance of the PV device (PCE < 1%) (summarized in Table 1). Moreover, the energy levels of pure Spiro-OMeTAD were badly mismatched with the tin-based perovskite, which induced a low device voltage [40,41,42]. 

### 2.2. Doping of Spiro-OMeTAD

#### 2.2.1. Spiro-OMeTAD + Li-TFSI + tBP

**Figure 2:**
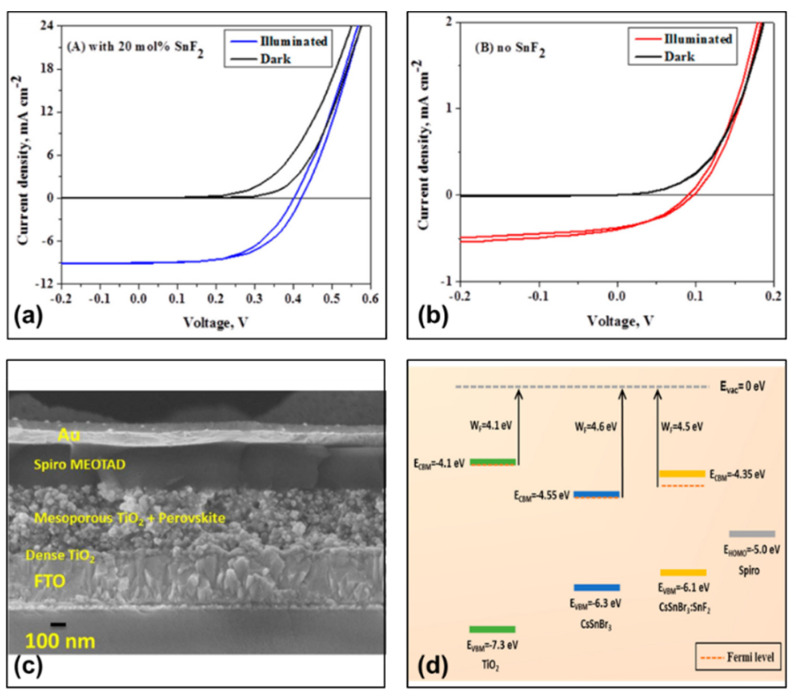
Current density–Voltage characteristics of the champion CsSnBr_3_ device (**a**) with SnF_2_ and (**b**) without SnF_2_ devices under dark and light conditions. (**c**) Cross–section scanning electron microscopy (SEM) image of CsSnBr_3_ devices. (**d**) Valence band maximum, conduction band minimum and Fermi energy levels of dense TiO_2_, CsSnBr_3_ and CsSnBr_3_ (with 20 mol% SnF_2_) materials [43]. Reprinted with permission from Ref. [42]. Copyright 2016 American Chemical Society.

Gupta et al. explored an all-inorganic tin-based perovskite photovoltaic device with Spiro-OMeTAD as the hole transport material and SnF_2_-doped cesium bromide tin (CsSnBr_3_) as the light absorbing layer. The device structure was FTO/d-TiO_2_/mp-TiO_2_/Perovskite/Spiro-OMeTAD/Au (Figure 2c) [43]. In their work, the Spiro-OMeTAD layer was prepared by adding 75 mg of Spiro-OMeTAD in 1 mL of chlorobenzene; followed by adding tBP and Li-TFSI to the solution to improve the conductivity by doping with tBP and Li-TFSI. Under simulated 100 mW/cm^2^ AM 1.5G irradiation, the device showed a power conversion efficiency of 2.10%, a short-circuit current of 9 mA/cm^2^, an open-circuit voltage of 410 mV and a fill factor of 58% (Table 1). In their work, 20 mol% SnF_2_ was doped in the light absorption layer. Figure 2a,b show the current density–voltage curves of the device (with/without SnF_2_) in the dark and under light, and it can be observed that the photovoltaic conversion efficiency is significantly improved by a factor of 200 with SnF_2_ doping. The addition of SnF_2_ dopant reduced the work function of CsSnBr_3_ (*WF* = 4.52 eV and *E*_VBM_ = −6.10 eV) and greatly lowered the ionization potential compared to the original CsSnBr_3_ (WF = 4.62 eV) and (*E*_VBM_ = −6.33 eV). The doping of Li-TFSI brings the highest occupied molecular orbital (HOMO) of the hole transport layer close to the EVBM of the perovskite (closer than that of pure spiro-OMeTAD), improving the energy level alignment between the tin-based perovskite layer and the hole transport layer, which in turn reduces the large voltage loss at the HTM/Perovskite interface (Figure 2d). The authors also point out that this conclusion needs further investigation. However, the lithium salts could bleach the perovskite film during the spiro-OMeTAD drop-casting process.

In summary, the conventional preparation methods of Spiro-OMeTAD and Li-TFSI present two main issues: First, the tin-based perovskite films were sensitive when exposed to acetonitrile solutions with tBP and Li-TFSI. The hygroscopic properties of the lithium salt in the Spiro-OMeTAD layer would accelerate the oxidation of Sn^2+^ to Sn^4+^, resulting in a sharp decrease in device performance. During the spin-coating of tBP:acetonitrile-doped Spiro-OMeTAD solution, the degradation of the tin-based perovskite film is unavoidable even with fast processing [41,43,44,45]. Chlorobenzene as a solvent alone or chlorobenzene with Spiro-OMeTAD:tBP does not change the color of the perovskite film; however, it may form a highly resistant interfacial phase after Spiro-OMeTAD deposition. The solar cells prepared by this method exhibit high–resistance characteristics, which would lead to a high voltage loss and consequently reduced fill factor [41]. Second, to achieve the champion performance of Spiro-OMeTAD, Li-TFSI is required to mediate the reaction of O_2_ with Spiro-OMeTAD in the presence of light to produce TFSI-stabilized radical cations with the lithium oxide to complete the doping. Such an oxidation process relies on the very slow entry and diffusion of oxygen through Spiro-OMeTAD:Li-TFSI; on the other hand, the tin-based perovskite film was extremely air-sensitive, which contrasted exactly against the oxidation process of Spiro-OMeTAD:Li-TFSI. The stability of encapsulated tin-based devices prepared using Spiro-OMeTAD:Li-TFSI:tBP as the hole transport layer was also relatively poor [39].

#### 2.2.2. Spiro-OMeTAD + H-TFSI + tBP 

**Figure 3:**
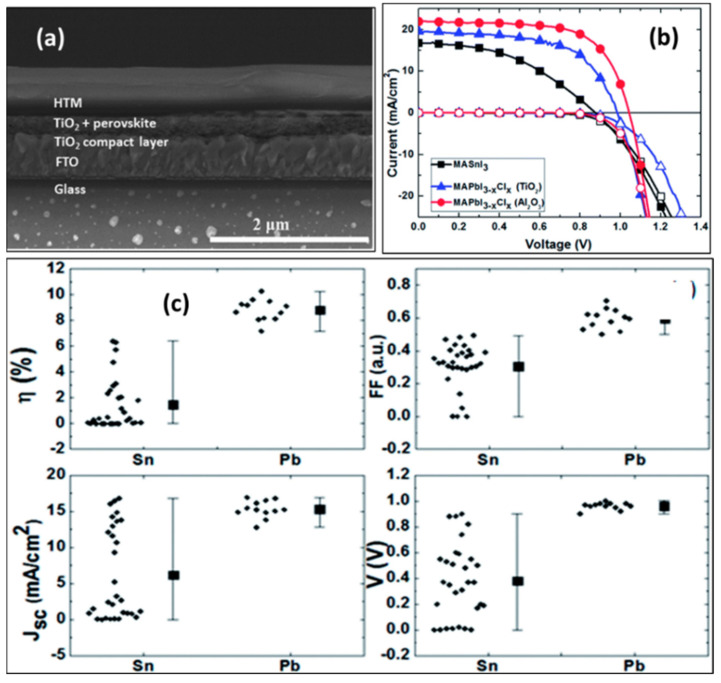
(**a**) Cross-sectional SEM image of the device with FTO/c-TiO_2_ (50 nm)/mp-TiO_2_/MASnI_3_ (400 nm)/Spiro-OMeTAD (doped, 600 nm)/Au. (**b**) Performance of the champion tin-based and lead-based perovskite solar cells from the same batch, with the bright current curves indicated by solid symbols and dark current curves shown with hollow symbols. (**c**) Current–voltage characteristics and device performance parameters for power conversion efficiency, fill factor, short-circuit current and open-circuit voltage, respectively. The device performance data are shown on the left side of the figure for tin-based MASnI_3_ and on the right side for lead-based MAPbI_3-x_ Cl_x_ for light-absorbing materials [25]. Reprinted with permission from Ref. [21]. Copyright 2014 The Royal Society of Chemistry.

To solve the problem whereby the hygroscopic property of lithium salts accelerates the oxidation of Sn^2+^ to Sn^4+^, Noel et al. used hydrogen bis(tri-fluoromethanesulfonyl) imide (H-TFSI) to replace Li-TFSI. With MASnI_3_ as the absorbing layer, they prepared FTO/c-TiO_2_/mp-TiO_2_/MASnI_3_/Spiro-OMeTAD/Au structure solar cells (Figure 3a) [25]. The formulation of the hole transport layer was 80 mM Spiro-OMeTAD, 10 mM H-TFSI and 80 mM tBP. The device obtained a photovoltaic conversion efficiency of 6.40%, an open-circuit voltage of 880 mV, a short-circuit current density of 16.80 mA/cm^2^ and 42% fill factor (Figure 3b, Table 1). The cell performance parameters using tin-based MASnI_3_ and lead-based (MAPbI_3-x_Cl_x_) as light absorber layer are shown in Figure 3c. The replacement of Li-TFSI by H-TFSI can partially solve the problem of the hygroscopic properties of lithium salts causing an acceleration in the oxidation of Sn^2+^ to Sn^4+^. However, another additive in the precursor solution of the cavity transport layer, tBP, can react with MASnI_3_ and form colorless coordination complexes or hydroxides. In addition, tBP, as a strong alkali, can also damage the film when used in large amounts or over a long period of time [47].

#### 2.2.3. Spiro-OMeTAD + H-TFSI + 2,6-lutidine

**Figure 4:**
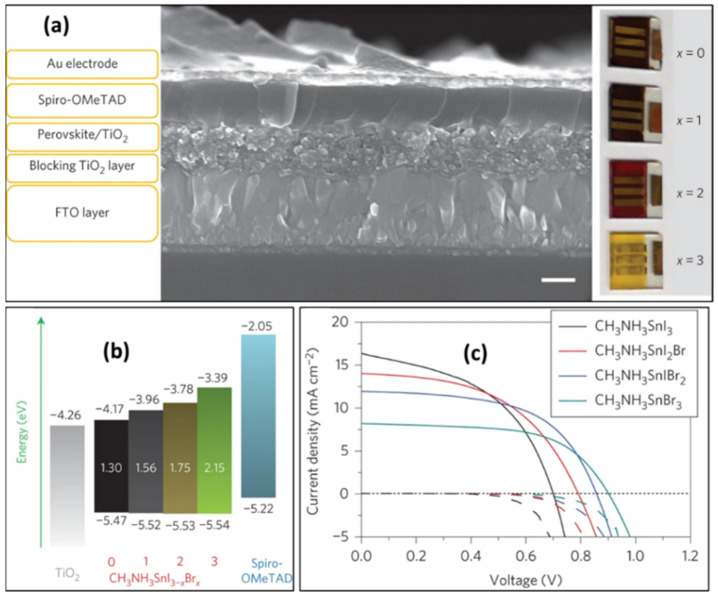
(**a**) SEM image of a tin-based perovskite solar cell with MASnI_3_ as the light absorbing layer; the structure of the device is FTO/c-TiO_2_/mp-TiO_2_/Perovskite/Spiro-OMeTAD/Au, and the real device image on the right side showing the color of the photovoltaic device made from MASnI_3-x_Br_x_. (**b**) Schematic of the energy level structure of a tin-based perovskite photovoltaic device; (**c**) Current–voltage diagram of tin-based photovoltaic device under one sunlight irradiation, MASnI_3_, MASnI_2_Br, MASnIBr_2_ and MASnBr_3_, respectively [26]. Reprinted with permission from Ref. [22]. Copyright 2014 Springer Nature.

In 2014, Hao et al. utilized MASnI_3-x_Br_x_ as the light absorbing layer and Spiro-OMeTAD with 2,6-lutidine replacement of tBP material (to enhance the hole mobility) as the hole transport layer, and prepared tin-based perovskite solar cells with device structure of FTO/c-TiO_2_/mp-TiO_2_/Perovskite/Spiro-OMeTAD/Au (Figure 4a) [26]. In this case, the preparation of the hole transport layer Spiro-OMeTAD was obtained from 72.3 mg of Spiro-OMeTAD, 30 mL of 2,6-lutidine, 17.5 μL of acetonitrile solution of Li-TFSI (concentration of 520 mg mL^−1^), dissolved in 1 mL of chlorobenzene solution. For the MASnI_3-x_Br_x_ perovskites, the conduction band changed from 4.17 eV for MASnI_3_, 3.96 eV for MASnI_2_Br, 3.78 eV for MASnIBr_2_, and 3.39 eV for MASnBr_3_. From the energy band alignment diagram, the change in the band gap (*E*_g_) of MASnI_3-x_Br_x_ is mainly due to the shift of the conduction band to an upper position. These energy level changes allow for band gap engineering and energetics tuning to achieve more efficient solar cell structures (Figure 4b). Ultimately, the device based on the MASnIBr_2_ obtained a maximum efficiency of 5.70%, corresponding to a short-circuit current of 12.30 mA/cm^2^, an open-circuit voltage of 820 mV and a fill factor of 57% (Figure 4c, Table 1). However, the 2,6-lutidine remained a base, and thus would damage the tin perovskite film over long time periods.

**Figure 5:**
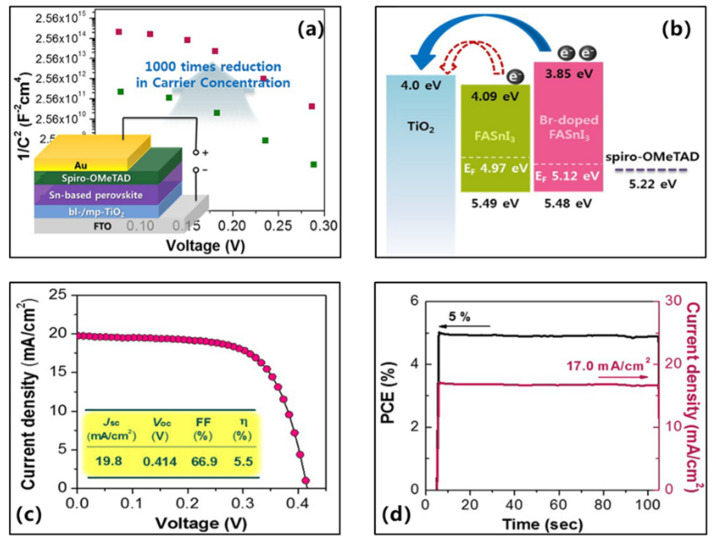
(**a**) Device diagram of FASnI_3_-based perovskite solar cell. (**b**) Energy schematics of TiO_2_, FASnI_3_ and Br-doped FASnI_3_ films. (**c**) The density–voltage curves of champion devices under reverse scan. (**d**) Stable efficiency and current–voltage curve of champion device measured at 293 mV voltage for 100s [46]. Reprinted with permission from Ref. [46]. Copyright 2018 American Chemical Society.

In 2017, Lee et al. in the preparation of Spiro-OMeTAD hole transport layer, they also replaced the tBP material with 2,6-lutidine. The device’s structure was FTO/c-TiO_2_/mp-TiO_2_/Perovskite/Spiro-OMeTAD/Au, with TiO_2_ as the electron transport layer and Br-doped (25 mol%) FASnI_3_ as the light-absorbing layer (Figure 5a) [46]. The precursor solution for the hole transport layer contained Spiro-OMeTAD/chlorobenzene (72.3 mg/mL, 1 mL) solution, 17.5 μL of H-TFSI/acetonitrile (509 mg/mL) solution and 30 μL of 2,6-lutidine. Br doping in FASnI_3_ significantly reduced the carrier density of the perovskite absorber (Figure 5a) and its interaction with the TiO_2_ electron energy levels of the transport layer were better matched (Figure 5b). The optimal cell device achieved a photovoltaic conversion efficiency of 5.50%, corresponding to a short-circuit current density of 19.80 mA/cm^2^, an open-circuit voltage of 414 mV, and a fill factor of 66.90% (Figure 5c, Table 1). In a 100-s maximum power point tracking measurement, the steady-state current density was 17.0 mA/cm^2^ and the stable power conversion efficiency was 5% (Figure 5d). Although the film color remained constant when 2,6-lutidine was used. However, since 2,6-lutidine itself is an alkali, it can still damage the film when used in a large amount or for a long period of time.

In summary, although efforts have been made to improve the performance of tin-based perovskite solar cell devices based on Spiro-OMeTAD layers, it is still challenging to obtain high-performance devices. This is because the use of dopants, whether tBP, or LiTFSi, or 2,6-lutidine always destroys the tin-based perovskite film; thus, the doped Spiro-OMeTAD cannot achieve high performance. Lessons could be learned from the improvement of electrical properties of Spiro-OMeTAD in Pb-based perovskite solar cells, such as organic Lewis acid [48]. In addition, based on this orthotropic structure of tin-based perovskite solar cells, there are also many laboratories that have conducted studies targeting Poly bis(4-phenyl)(2,4,6-trimethylphenyl) amine (PTAA), poly 3-hexylthiophene (P3HT), four tetraphenylethylene (TPE), and other hole transport materials.

## 3. Replacement of Organic Materials for Spiro-OMeTAD

### 3.1. Organic PTAA

#### 3.1.1. PTAA + LiTFSI + tBP

**Figure 6:**
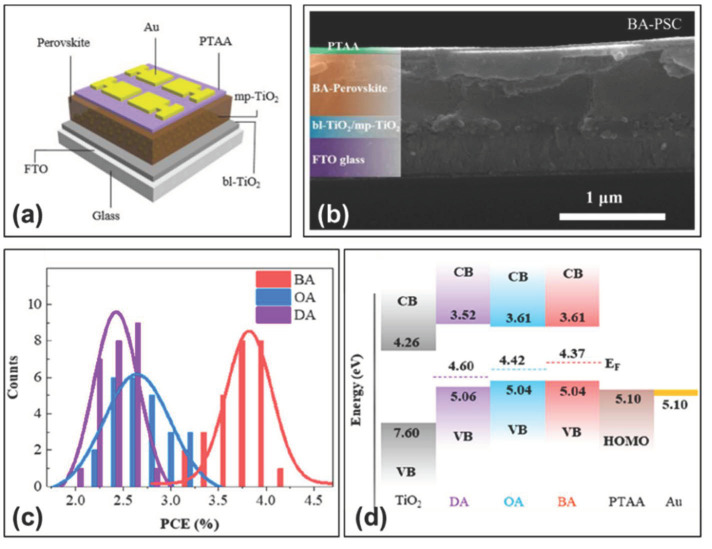
(**a**) Device diagram based on BA-perovskite thin film; (**b**) cross-sectional SEM image of 2D/3D perovskite solar cells; (**c**) PCE histogram of 2D/3D perovskite solar cells with 20 samples. (**d**) Schematic diagram of the energy level structure of 2D/3D perovskite solar cells [49]. Reprinted with permission from Ref. [47]. Copyright 2020 American Chemical Society.

In 2020, Li et al. utilized 2D tin-based Ruddlesden-Popper (R-P) phase perovskites of A_2_(FA)_n-1_SnnI_3n+1_(A=BA=CH_3_(CH_2_)_3_NH_3_^+^, or=OA=CH_3_(CH_2_)_7_NH^+^, or=DA=CH_3_(CH_2_)_11_NH_3_^+^) as the light-absorbing layer, and utilized PTAA as the hole transport material to fabricate the perovskite solar cells (FTO/c-TiO_2_/mp-TiO_2_/BA-PVSK/PTAA/Au) [49]. The PTAA precursor solution consisted of 10 mg of PTAA, 10 μL of Li-TFSI in acetonitrile (170 mg/mL) and 5 μL of tBP in 1 mL of toluene solution. Ultimately, the device obtained a power conversion efficiency of 4%, a short-circuit current of 24 mA/cm^2^, an open-circuit voltage of 420 mV, a fill factor of 40.20% (Figure 6a,b, Table 2) and a higher average PCE relative to the organic spacer cations with different chain lengths (Figure 6c). The effect of the organic spacer cations with different chain length on the crystal orientation and phase distribution of perovskite films was investigated. An increase in chain length would decrease the Fermi energy level EF, and the valance band maximum of these 2D perovskite were estimated as 5.04 eV for BA, 5.04 eV for OA, and 5.06 eV for DA, which were well matched with the PTAA hole transport layer (5.1 eV) (Figure 6d). The organic spacer cations with shorter alkyl chain lengths (e.g., BA) would promote vertical crystal growth of perovskite films, which could reduce Sn^2+^ oxidation and improved charge carrier transport.

To investigate the doping-effect difference between Spiro-OMeTAD + Li-TFSI + tBP and PTAA + LiTFSI + tBP, Handa et al. investigated the near-band-edge optical response devices based on FTO/c-TiO_2_/mp-TiO_2_/MASnI_3_/HTL/Au structure and the source of voltage loss in the devices [50,51]. The methods used to prepare the hole transport layer were all toluene solution of hole transport materials with Li-TFSI (acetonitrile) solution and tBP additives. The photovoltaic parameters extracted after 20 days of preparation were as follows: 26.10 mA/cm^2^ short circuit current density, 300 mV open-circuit voltage, 30% fill factor. The short-circuit current density of the device was slightly higher than that of its lead-based counterpart (24 mA/cm^2^), since MASnI_3_ perovskite can absorb more photons in the near-infrared region up to 1000 nm. However, the photoconversion efficiency is about 2.30%, because the open-circuit voltage was only 230 mV and the fill factor was only 39% (Table 2). The replacement of Spiro-OMeTAD with PTAA did not fundamentally solve the issue of additive use on the tin-based perovskite films, and the HOMO energy level of PTAA (−5.10 eV) does not match the valence band energy level of MASnI_3_ (−4.75 eV), induced an energy barrier for the hole carrier transport.

#### 3.1.2. PTAA:4-Isopropyl-4′-methyldiphenyliodonium Tetrakis (Pentafluorophenyl)borate (TPFB)

**Figure 7:**
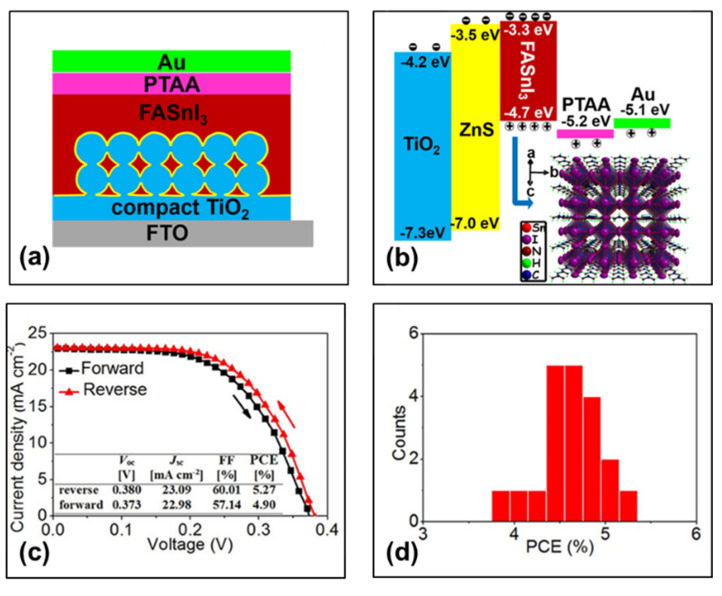
(**a**) Schematic diagram of the device structure and (**b**) energy band diagram of the FASnI_3_ solar cell and crystal structure of the perovskite light absorber. (**c**) Current density–voltage curves of the champion performing FASnI_3_ solar cell using mesoporous TiO_2_-ZnS measured at reverse and forward voltage scans. (**d**) PCE histograms of 20 FASnI_3_ solar cells using mesoporous TiO_2_ -ZnS measured under reverse voltage scan [52]. Reprinted with permission from Ref. [50]. Copyright 2016 American Chemical Society.

In 2016, with TPFB as the hole transport layer, Ke et al. prepared tin-based perovskite photovoltaic devices with FTO/c-TiO_2_/mp-TiO_2_/FASnI_3_/PTAA:TPFB/Au structure (Figure 7a) [52]. The TiO_2_-ZnS electron transfer layer effectively facilitates electron extraction from FASnI_3_ into TiO_2_, resulting in devices with efficient electron transfer and lower interface recombination. The champion performing perovskite solar cell achieved a photovoltaic conversion efficiency of 5.27% with an open-circuit voltage of 380 mV, a short-circuit current density of 23.09 mA/cm^2^, and a fill factor of 60% (Figure 7c and Table 2). Figure 7d shows that the perovskite solar cell exhibits good reproducibility and high average performance cells. The hole transport material consists of 32 mg PTAA and 3.6 mg TPFB in 1.6 mL chlorobenzene solution. Among them, the valence band maximum of FASnI_3_ (−4.7 eV) is slightly higher than that of PTAA (−5.2 eV), which may present a barrier to hole carrier transport (Figure 7b). However, the FASnI_3_ film is briefly exposed to air during device fabrication, so the surface of the perovskite film is p-type doped and forms an ohmic contact with the PTAA layer. Therefore, the hole carriers can be efficiently transferred from perovskite FASnI_3_ to hole transport PTAA layer.

**Figure 8:**
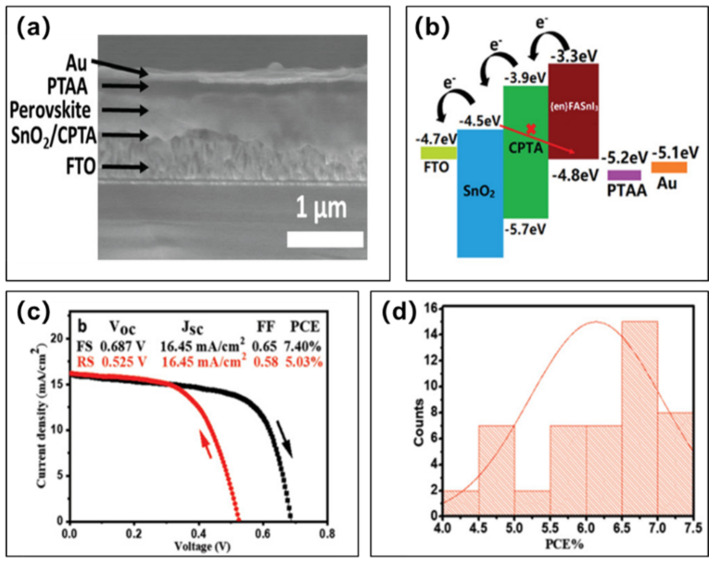
(**a**) Cross-sectional SEM image of the tin-based perovskite solar cell. (**b**) Energy band diagram of the tin-based perovskite solar cell. The red arrows illustrate the complex of the SnO_2_/en-FASnI_3_ interface. (**c**) Current density–voltage curves of the champion device; the open-circuit voltages for forward and reverse scans were 687 mV and 525 mV (50 mV/s scan rate), respectively. (**d**) Photovoltaic conversion efficiency histograms for 48 solar cells [58]. Reprinted with permission from Ref. [56]. Copyright 2019 John Wiley and Sons.

Subsequent research groups have conducted several investigations on tin-based perovskite solar cells based on the hole transport layer of PTAA:TPFB [47,53,54,55,56,57,58]. Among them, Yang et al. used PTAA+TPFB as the hole transport layer, en-FASnI_3_ as the light-absorbing layer, SnO_2_-C_60_ pyrrolidine tris-acid (CPTA) as the electron transport layer, and en-FASnI_3_ to fabricate the tin-based perovskite solar cells FTO/SnO_2_-CPTA/en-FASnI_3_/PTAA:TPFB/Au (Figure 8a,b) [58]. Similar to the FASnI_3_ perovskite layer, the valance band maximum of en-FASnI_3_ was also higher than HOMO of PTAA; however, the surface of en-FASnI_3_ perovskite film was p-doped, producing the ohmic contact between them. The champion device achieved a photovoltaic conversion efficiency of 7.40%, an open-circuit voltage of 720 mV, a short-circuit current density of 16.45 mA/cm^2^, and a fill factor of 65% (Figure 8c). Meanwhile, the reproducibility of the devices was excellent, with most of them having an efficiency of more than 6% (Figure 8d).

In summary, the combination of the PTAA and TPFB as hole transport material showed great potential in the inverted FASnI_3_-based solar cells, in which the perovskite layer is exposed to air during the preparation of FASnI_3_ to form surface p-type doping, so they form an ohmic contact with PTAA layer, which effectively minimized the charge carrier transport caused by the energy-level mismatch between the hole transport layer and the tin-based perovskite layer problem. Compared with Spiro-OMeTAD, Spiro-OMeTAD still failed to achieve good performance despite the use of different kinds of additives. In contrast, the PTAA cavity transport layer uses the additive TPFB, and the en-FASnI_3_-based device achieved a high performance.

### 3.2. Organic P3HT

**Figure 9:**
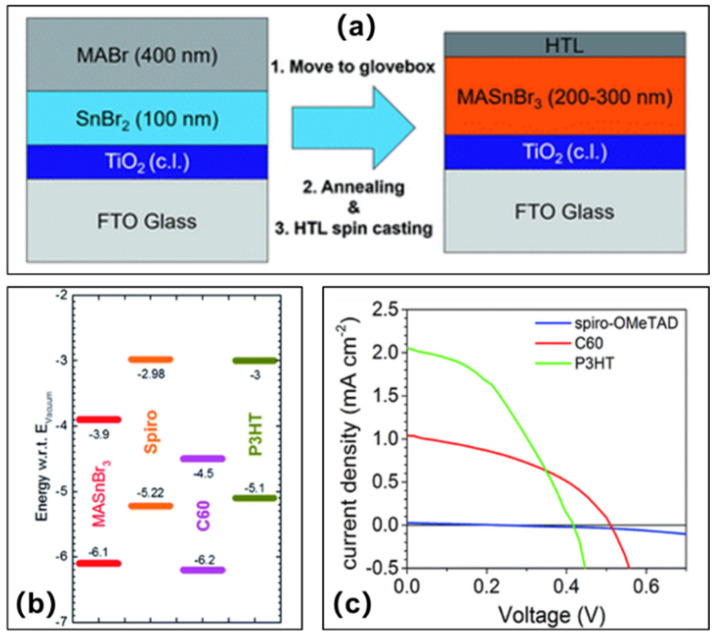
(**a**) Schematic diagram of tin-based perovskite solar cell device preparation process; the first layer is 100 nm SnBr_2_, and the second layer is 400 nm MABr to avoid any air contact with elemental Sn. (**b**) Energy level diagram of MASnBr_3_ perovskite and hole transport materials. (**c**) Current density–voltage curves of MASnBr_3_ perovskite solar cells fabricated by co-evaporation of SnBr_2_ and MABr and with Spiro-OMeTAD (blue), C_60_ (red) and P3HT (green) [45]. Reprinted with permission from Ref. [44]. Copyright 2016 The Royal Society of Chemistry.

Jung et al. utilized SnBr_2_ and MABr for co-evaporation of lead-free MASnBr_3_ perovskite crystal films (Figure 9a), and utilized three hole-transport materials Spiro-OMeTAD, C_60_ and P3HT as the hole transport layer. They finally fabricated the tin-based perovskite solar cells FTO/c-TiO_2_/MASnBr_3_/HTM/Au [45]. The band gaps and energy levels of MASnBr_3_ perovskite and the hole transport materials were measured based on the results of ultraviolet photoelectron emission spectroscopy and ultraviolet–visible absorption spectroscopy (Figure 9b). The devices prepared with Spiro-OMeTAD showed a power conversion efficiency of 0.002%, a short-circuit current density of 0.03 mA/cm^2^, an open-circuit voltage of 236 mV, and a fill factor of 25.6%. The power conversion efficiency of the device with C_60_ was 0.22%, the short-circuit current density was 1.04 mA/cm^2^, the open-circuit voltage was 509 mV, and the fill factor was 80%. The devices prepared with P3HT showed a power conversion efficiency of 0.35%, a short-circuit current density of 2.05 mA/cm^2^, an open-circuit voltage of 415 mV, and a fill factor of 41%. The highest power conversion efficiency and short-circuit current density were observed in the devices with P3HT as hole transport layer (Figure 9c). Part of the current is provided by the additional photogenerated charge of P3HT. On the other hand, a higher efficiency of up to 1.12% was obtained using P3HT as HTL when we used sequential deposition, corresponding to an open-circuit voltage of 498 mV and a short-circuit current density of 4.27 mA/cm^2^, with a fill factor of 49.10% (Table 2). Due to the low valance band level of MASnBr_3_ perovskite, C60 (−6.2 eV) becomes the optimal candidate as hole transport layer among them. In addition, as expected, the open-circuit voltage of C60-based device was higher than that of P3HT (−5.1 eV)-based devices.

**Figure 10:**
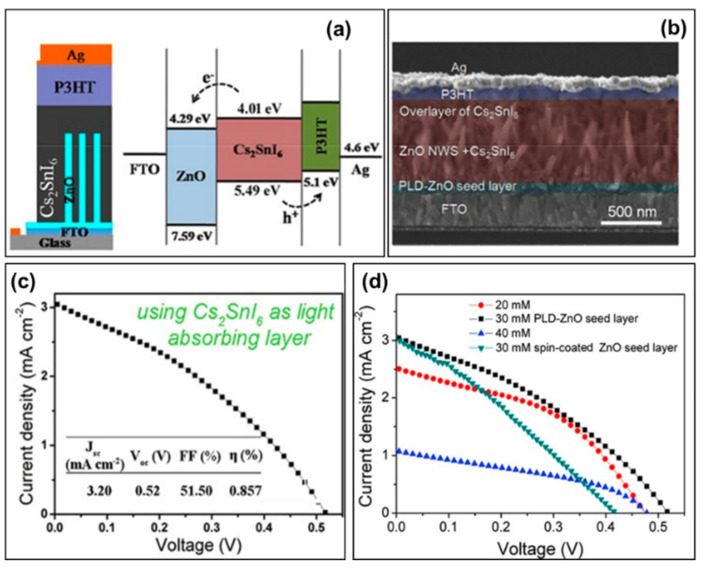
(**a**) Device and energy band structure of FTO/ZnO dense layer/ZnO nanorods/Cs_2_SnI_6_/P3HT/Ag solar cell. (**b**) Cross-sectional SEM images of the device structure. (**c**) Current density–voltage curves of the solar cell based on Cs_2_SnI_6_. (**d**) J-V curves of Cs_2_SnI_6_ perovskite solar cells with ZnO nanorods grown on different seed layers and different precursors [59]. Reprinted with permission from Ref. [57]. Copyright 2016 John Wiley and Sons.

In 2016, with P3HT as hole transport materials, Qiu et al. prepared mesoporous solar cells with FTO/c-ZnO/n-ZnO/Cs_2_SnI_6_/P3HT/Ag structures using air-stable perovskite Cs_2_SnI_6_ as the light-absorbing material (Figure 10a,b) [59]. In this case, the P3HT precursor solution was prepared by dissolving 10 mg of P3HT in 1 mg of chlorobenzene; and the devices with ZnO nanorods grown in the PLD-ZnO seed layer showed improved performance than those with spin-coated seed layer (Figure 10d), finally obtaining a power conversion efficiency of nearly 1%, a short-circuit current density of 3.20 mA/cm^2^, an open-circuit voltage of 520 mV and a fill factor of 51.50% (Figure 10c,d, Table 2). The energy-level alignments of conduction and valance bands of the Cs_2_SnI_6_ perovskite allow for the efficient injection of electrons into the electron transport layer and transport of holes to the P3HT layer.

**Figure 11:**
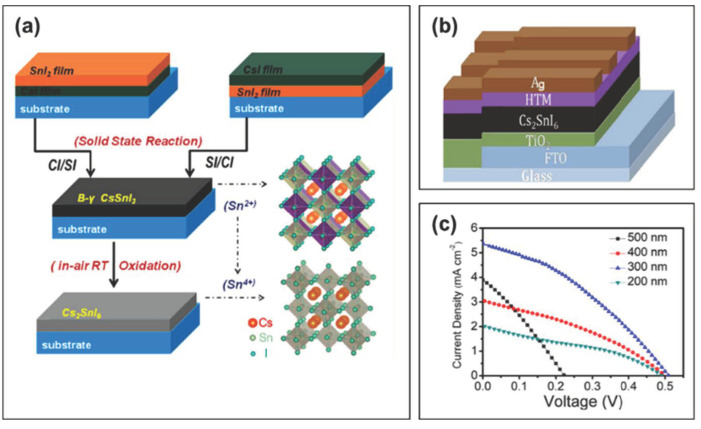
(**a**) Schematic diagram of the growth of Cs_2_SnI_6_ film from CsSnI_3_ by a two-step deposition method; the perovskite crystal structures of Cs_2_SnI_6_ and CsSnI_3_ at the bottom right of the figure. (**b**) Schematic diagram of the structure of Cs_2_SnI_6_-based perovskite solar cells. (**c**) Current density–voltage curves of perovskite solar cells fabricated based on Cs_2_SnI_6_ with different thicknesses [60]. Reprinted with permission from Ref. [58]. Copyright 2017 Elsevier.

In 2017, Qiu et al. utilized B-γ-CsSnI_3_ that spontaneously oxidizes to air-stable Cs_2_SnI_6_ and prepared FTO/TiO_2_/Cs_2_SnI_6_/P3HT/Ag-structured perovskite solar cells (Figure 11a,b) [60]. By optimizing the thickness of the perovskite absorber layer, a power conversion efficiency of about 1% was achieved at an open-circuit voltage of 510 mV and a short-circuit current of 5.41 mA/cm^2^ (Figure 11c, Table 2). For the devices with all-inorganic perovskite material Cs_2_SnI_6_ as the light-absorbing layer, among the hole transport layers of Spiro-OMeTAD, C_60_ and P3HT, the devices with P3HT as the hole transport layer exhibited the highest power conversion efficiency and short-circuit current density. This can be explained by the HOMO of P3HT (−5.10 eV) matching well with the valence band of the all-inorganic perovskite material Cs_2_SnI_6_ (−5.49 eV). The device performance decreased thereafter and failed after about one month. The organic hole transport material P3HT was considered as one of most problematic sources responsible for the performance loss.

### 3.3. Organic Small Molecule TPE

**Figure 12:**
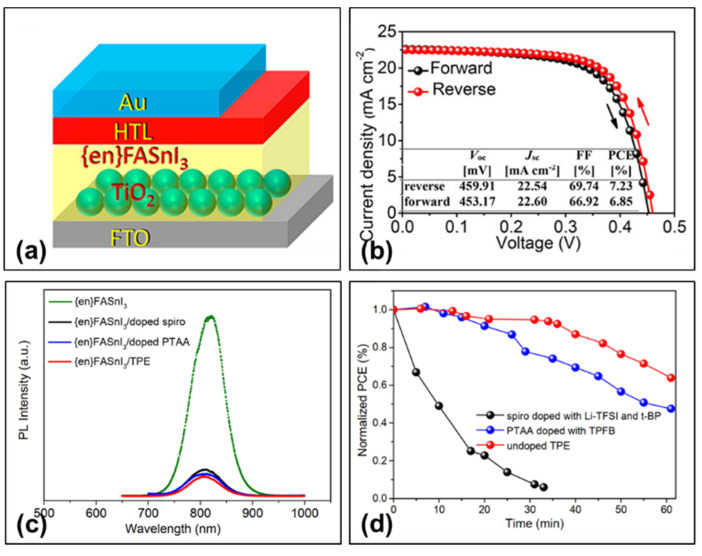
(**a**) Device structure of TPE-based perovskite solar cell. (**b**) Current density–voltage curves of the champion perovskite solar cell under reverse and forward voltage scans. (**c**) Photoluminescence spectra of perovskite films with different hole transport layers. (**d**) Aging tests of unencapsulated solar cells with various hole transport layers in ambient air [41]. Reprinted with permission from Ref. [40]. Copyright 2018 American Chemical Society.

**Figure 13:**
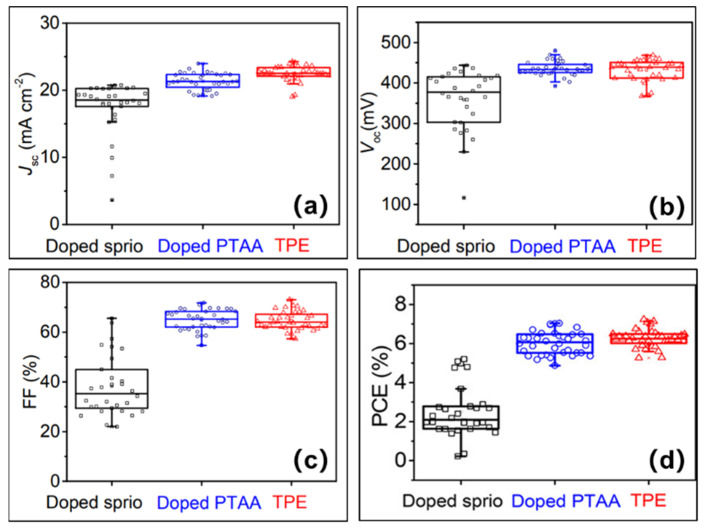
Statistics of (**a**) short-circuit current, (**b**) open-circuit voltage, (**c**) fill factor and (**d**) power conversion efficiency for FASnI_3_-based solar cells with different hole transport layers [41]. Reprinted with permission from Ref. [40]. Copyright 2018 American Chemical Society.

In 2018, Ke et al. synthesized a novel small molecule hole transport layer material tetra-triphenylamine (TPE), which is a compound with excellent physicochemical properties, such as easy dissolution in common organic solvents to form uniform films [41]. The mobility was comparable to that of doped Spiro-OMeTAD and PTAA: 1.4 ± 0.1 × 10^−3^, 1.3 ± 0.1 × 10^−3^, and 1.6 ± 0.2 × 10^−3^ cm^2^V^−1^s^−1^, respectively. The HOMO energy level was −4.99 eV and LUMO energy level was −2.22 eV, which match well with the tin-based perovskite energy level. In addition, the TPE exhibited good thermal stability with high decomposition temperature of 425 °C. The tin-based perovskite solar cell with FTO/c-TiO_2_/mp-TiO_2_/en-FASnI_3_/TPE/Au was prepared (Figure 12a). TPE precursor solution was prepared by adding 5 mg of TPE in a mixture of 0.5 mL of chlorobenzene and 0.5 mL of chloroform. As shown in Figure 12c, the TPE-coated perovskite film showed a good quenching effect, implying that the photogenerated carriers were more efficiently transported into the TPE carrier transport layer. As a result, the good hole transport ability of TPE allowed the solar cell to have a high short-circuit current density and fill factor and the champion stability (Figure 12d). Finally, the solar cell exhibited a power conversion efficiency of 6.85%, an open-circuit voltage of 453 mV, a short-circuit current density of 22.60 mA/cm^2^, and a fill factor of 67% measured under the forward voltage scan. Under the reverse scan, the power conversion efficiency was 7.23%, the open-circuit voltage was 459 mV, the short-circuit current density was 22.54 mA/cm^2^, and the fill factor was 70%. Moreover, the hysteresis was quite small (Figure 12b). Since TPE had several advantages such as low cost, easy preparation, well-matched tin-based perovskite energy levels, high mobility and excellent thermal stability, etc., it is well suited to be used as a superior hole transport material for the development of economical and high-performance tin-based perovskite solar cells. The devices using the TPE as the hole transport layer had achieved improved performance compared to that of Spiro-OMeTAD- and PTAA-doped devices, even without adding any additives or dopants in TPE, which showed great potential for the fabrication of low-cost, high-efficiency and large-scale tin-based perovskite solar cells (Figure 13).

In summary, regarding the use of PTAA + Li-TFSI + tBP, there are easily caused issues that arise from the incorporation of dopants. This is not to say that all dopants will destroy the perovskite film, with TPFB used as the dopant in PTAA layer, it still obtains a good performance. The FASnI_3_-based perovskite solar cells eventually achieved a 7.40% power conversion efficiency and 720 mV open-circuit voltage. For undoped P3HT, in Cs_2_SnI_6_-based perovskite solar cells, the power conversion efficiency and short-circuit current density in P3HT-based devices were higher than those in Spiro-OMeTAD and C_60_-based devices. In addition, for the undoped TPE, in FASnI_3_-based perovskite solar cells, the short-circuit current density and open-circuit voltage were higher than those in doped Spiro-OMeTAD and C_60_-based device. However, Spiro-OMeTAD and doped PTAA-based devices showed small hysteresis and quite good reproducibility. In general, the HOMO level of the perovskite layer matches well with that of the hole transport layer. The HOMO level of tin-based perovskite is slightly deeper than that of hole transport materials, which is favorable for charge carrier transport. For example, the HOMO energy level of P3HT is −5.10 eV, which matches well with that of Cs_2_SnI_6_ energy level (−5.49 eV); TPE (−4.99 eV) and en-FASnI_3_ (−4.80 eV), which also matched well. TPE has a shallower energy level than that of Spiro-OMeTAD and PTAA, which means they have a lower energy mismatch; thus, a higher short-circuit current density and open-circuit voltage can be achieved.

### 3.4. Commonly Used Hole Transfer Material Poly(3,4-thylenedioxythiophene): Polystyrene Sulfonate (PEDOT:PSS)

Although the performance of tin-based perovskite photovoltaic devices with regular device structure has been improved in the past few years, from the perspective of the device preparation process: (i) The quality of tin-based perovskite films deposited on electron transport layers (e.g., TiO_2_, C_60_, ZnO, and ZrO) is poor, which leads to poor interfacial contacts and instability issues in photovoltaic devices. (ii) The use of additives in hole transport layer on the tin-based perovskite layer would damages the perovskite film. Thus, obtaining high-performance devices with a regular structure is still challenging. While inverted tin-based solar cell devices can avoid these problems to some extent.

**Figure 14:**
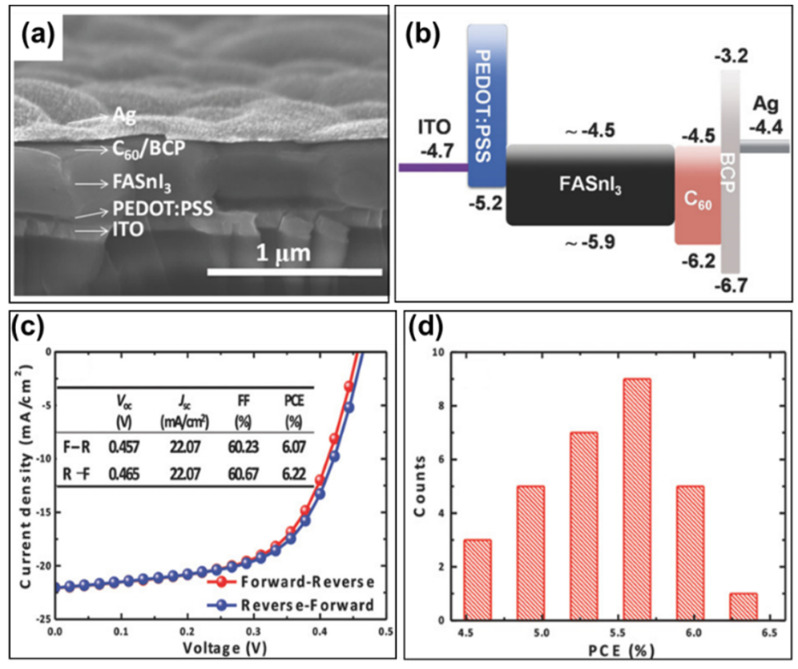
FASnI_3_-based inverted perovskite solar cell: (**a**) Cross-sectional SEM image of the device. (**b**) Schematic diagram of the energy levels. (**c**) Current density–voltage curve characteristics at AM1.5G illumination under reverse and forward scans. (**d**) Power conversion efficiency histograms of 30 solar cell devices with 10% SnF_2_ additive [61]. Reprinted with permission from Ref. [59]. Copyright 2016 John Wiley and Sons.

In inverted structured tin-based perovskite solar cells, PEDOT:PSS is the most commonly used hole transport layer material [61,62,63,64,65,66,67,68,69,70,71,72,73,74,75,76,77,78,79,80,81,82,83,84,85,86,87,88,93,94]. In 2016, Liao et al. used PEDOT:PSS as the hole transport layer and synthesized high-quality, uniform, full-coverage FASnI_3_ perovskite films using SnF_2_ as additive. The SnF_2_ additive inhibits the oxidation of Sn^2+^ in tin-based perovskites and reduces the hole density in the synthesized films [61]. Moreover, the spin-coating of diethyl ether on PEDOT:PSS/ITO substrate produces a more uniform and complete coverage of the perovskite films. Finally, they prepared inverted devices with the structure of ITO/PEDOT:PSS/FASnI_3_ + 10 mol% SnF_2_/C_60_/BCP/Ag (Figure 14a,b). The device achieved a power conversion efficiency of 6.22%, an open-circuit voltage of 465 mV, a short-circuit current density of 22.07 mA/cm^2^ and a fill factor of 60%. Meanwhile, the device exhibited a negligible current density–voltage hysteresis behavior under the forward and reverse scan (Figure 14c, Table 2). Among the 30 devices fabricated in the same batch, the average power conversion efficiency was 5.41 ± 0.46%, the average open-circuit voltage was 449 ± 23 mV, the average short-circuit current density was 20.69 ± 0.95 mA/cm^2^, and the average fill factor was 58.20 ± 2.50% (Figure 14d) with good reproducibility. After the reports of Snaith et al. and Kanatzidis et al. in 2014 [25,26], Liao’s work was considered as another substantial improvement in the power conversion efficiency of tin-based perovskite solar cells. The PEDOT:PSS was used as the anode buffer layer and the hole transport layer to transport holes from the perovskite to the ITO anode, but the HOMO energy level of the PEDOT:PSS is slightly higher in the energy diagram shown in Figure 14b. The light-intensity-dependence measurement suggested that the open-circuit voltage was limited by the imbalanced charge carrier transport at the HTL/FASnI_3_ and FASnI_3_/ETL interface, which was not ideal for optimal device performance, and more work was needed in hole transport layer optimization and interface engineering, to further improve the performance of Sn-based perovskite solar cells.

**Figure 15:**
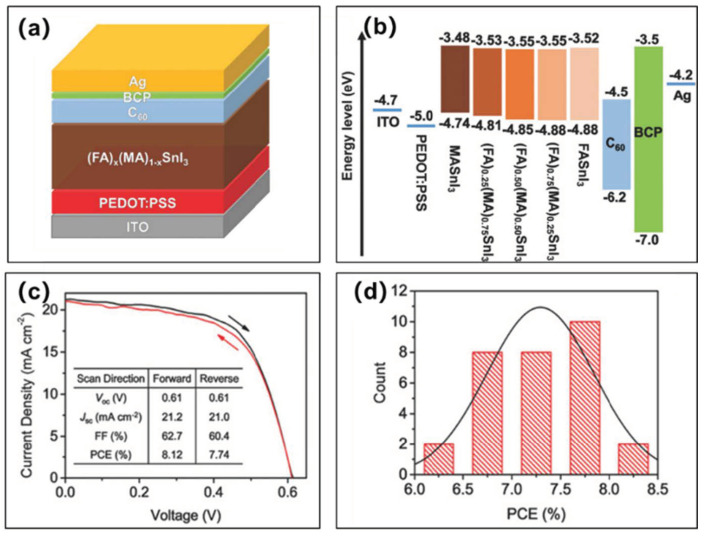
(**a**) Schematic diagram of the device structure. (**b**) Device energy level diagram. (**c**) Current density–voltage curves of the champion devices measured in forward and reverse scan. (**d**) Power conversion efficiency histograms from multiple batches [64]. Reprinted with permission from Ref. [62]. Copyright 2017 John Wiley and Sons.

Subsequently, with PEDOT:PSS as the hole transport layer and with mixed FA and MA cations (FA)_x_(MA)_1−x_SnI_3_ perovskite material as the light absorber layer, Zhao et al. fabricated solar cell devices with the structure of ITO/PEDOT:PSS/(FA)_x_(MA)_1−x_SnI_3_/C_60_/BCP/Ag (Figure 15a) [64]. The final device achieved a maximum power conversion efficiency of 8.12% (7.74%, reverse scan), corresponding to a short-circuit current density of 21.2 (21.0, reverse scan) mA/cm^2^, an open-circuit voltage of 610 (610, reverse scan) mV, a fill factor of 62.70% (60.40%, reverse scan), and a negligible hysteresis (Figure 15c and Table 2). The surface of PEDOT:PSS is treated by organic ammonium salts in the perovskite, thus reducing the work function of PEDOT:PSS (−5.0 eV) during perovskite deposition, which was around −4.7 eV. In other words, the treatment mitigated the mismatch between the valence band of tin-based perovskite (−4.80 eV) and HOMO of PEDOT:PSS, since the work function was more or less lowered during the perovskite deposition (Figure 15b). In the forward scan mode, the average power conversion efficiency of the 30 devices was 7.29 ± 0.55%, the average open-circuit voltage was 570 ± 20 mV, the average short-circuit current density was 20.70 ± 0.60 mA/cm^2^, and the average fill factor was 61.60 ± 2.50%, which showed a high reproducibility during the device fabrication (Figure 15d). 

**Figure 16:**
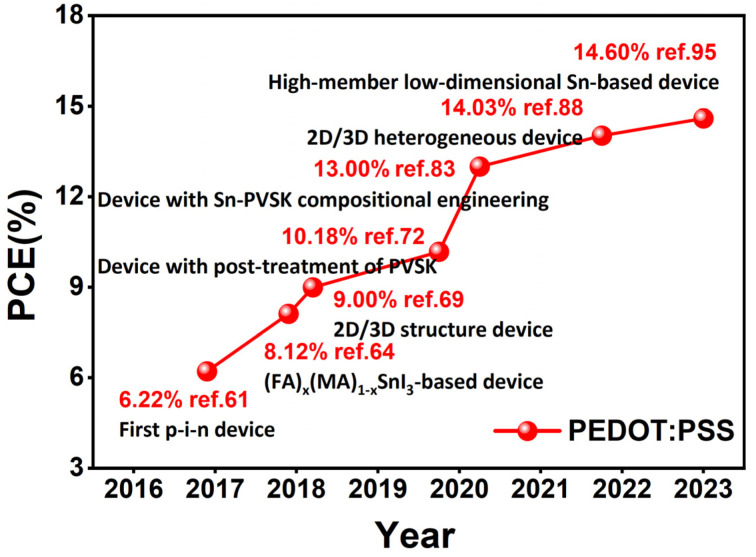
Efficiency development diagram of tin-based perovskite solar cells with pure PEDOT:PSS as hole transport layer [61,64,69,72,83,88,95].

In 2021, with PEDOT:PSS as the hole transport layer, and with 2D/3D structure phenethylammonium bromide (FPEABr)/FASnI_3_ as the perovskite light-absorption layer, Yu et al. fabricated the device ITO/PEDOT:PSS/Perovskite/ICBA/BCP/Al. The unique 2D/3D structure effectively suppressed the oxidation of Sn^2+^ to Sn^4+^ and reduced the defect density. Moreover, the 2D layer at the perovskite/PEDOT:PSS interface inhibits the migration of iodine ions from tin perovskite to the hole transport material PEDOT:PSS layer [88]. Finally, the device achieved a certified efficiency of 14.03%, corresponding to an open-circuit voltage of 828 mV, a short-circuit current of 24.50 mA/cm^2^, and a fill factor of 69.40% (Table 2). The 2D/3D tin-based perovskite films have a shallower valence band value (−5.05 eV) than that of FASnI_3_ (−5.15 eV) film, which matches better with the adjacent PEDOT:PSS layer (−5.0 eV). This will enhance the charge carrier extraction at the perovskite/PEDOT:PSS interface and improve the device performance. In addition to these groups mentioned above, there are several other research groups who have employed PEDOT:PSS as the hole transport material and also achieved excellent performance. The device-efficiency records of their work are shown in Figure 16.

Typically, the work function of PEDOT:PSS is −5.20 eV; however, the work function of PEDOT:PSS can be modified. When the surface of PEDOT:PSS is treated by the organic ammonium salt FA_0.75_MA_0.25_SnI_3_(−4.80 eV), the work function of PEDOT:PSS was modified to −5.0 eV, which reduces the energy-level mismatch between them. The HOMO energy level of the PEDOT:PSS layer should be higher than that of the perovskite layer, but the energy gap between them should not too high; for example, the HOMO gap between PEDOT:PSS (−5.20 eV) and FASnI_3_ (−5.90 eV) is too high, which is not ideal for device performance.

#### 3.4.1. Polyethylene Glycol (PEG)-PEDOT:PSS 

**Figure 17:**
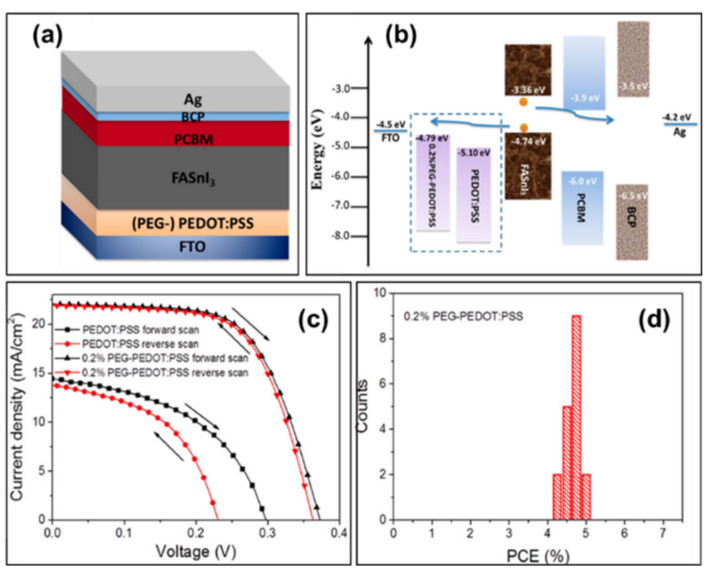
(**a**) Schematic diagram of the device structure. (**b**) Device energy level diagram. (**c**) Current density–voltage curves of devices with PEDOT:PSS and 0.2% PEG-PEDOT:PSS as the hole transport layer. (**d**) Power conversion efficiency histogram of 18 FASnI_3_ devices based on 0.2% PEG-PEDOT:PSS [89]. Reprinted with permission from Ref. [90]. Copyright 2018 American Chemical Society.

**Figure 18:**
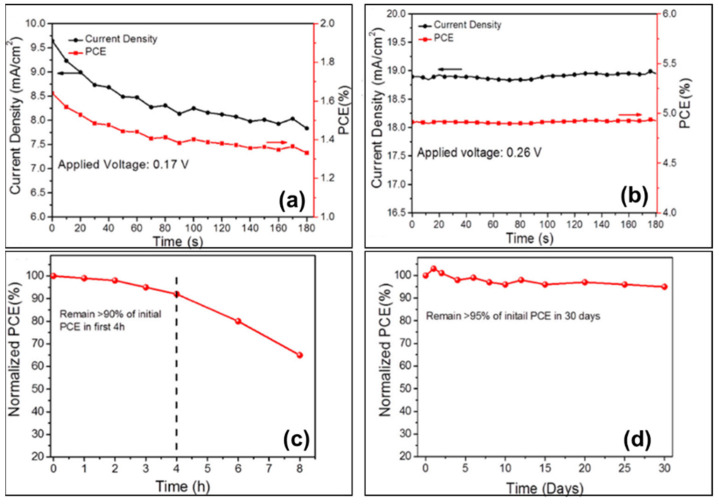
Steady-state current density and stable power conversion efficiency of devices based on (**a**) PEDOT:PSS and (**b**) 0.2% PEG-PEDOT:PSS under continuous AM 1.5G illumination, devices based on 0.2% PEG-PEDOT:PSS were tested for device stability during storage (**c**) under ambient conditions and (**d**) in a glove box filled with N_2_ [89]. Reprinted with permission from Ref. [90]. Copyright 2018 American Chemical Society.

The current density–voltage hysteresis in perovskite solar cells presents a serious issue because it affects the power conversion efficiency and stability of the devices. In a previously conducted experiment, 0.05%, 0.1% and 0.2% (volume ratio) PEG-400 were added to an aqueous solution of PEDOT:PSS, and the devices with the structure of FTO/(PEG-)PEDOT:PSS/FASnI_3_/PCBM/BCP/Ag were prepared (Figure 17a) [89]. The devices based on 0.2% PEG-PEDOT:PSS showed the champion performance with power conversion efficiencies of 5.12% and 5.03%; open-circuit voltages of 370 mV and 360 mV, short-circuit current densities of 22.06 mA/cm^2^ and 21.90 mA/cm^2^, and the fill factor of 62.70% and 63.80% in the forward and reverse scan, respectively (Table 2). A significant reduction in hysteresis was also shown, in addition to high reproducibility and an improved device stability with 4.91% stable efficiency (Figure 17c,d and Figure 18). The concentration of PEG could modulate the work function of this hole transport layer, thus reducing the energy-level mismatch between the hole transport layer and the tin-based perovskite layer; the valence band of FASnI_3_ is −4.74 eV and the work function of PEDOT:PSS is −5.10 eV, with an energy level mismatch of about 0.36 eV between them. After 0.2% PEG-doping in PEDOT:SS, the energy-level mismatch between FASnI_3_ and PEG-PEDOT:PSS was decreased to 0.05 eV (Figure 17b).

Moreover, with PEG-PEDOT:PSS as the hole transport layer, in 2020, Liu et al. prepared thin-based perovskite solar cells with the structure ITO/PEG-PEDOT:PSS/TG-FASnI_3_/C_60_/BCP/Ag [90]. The highly crystalline TG-FASnI_3_ films were prepared by pre-treating FASnI_3_ perovskite films with organic halide salt n-propylammonium iodide (PAI) solution prior to annealing. The device achieved a certified stable efficiency of 11.22%, corresponding to an open-circuit voltage of 695 mV, a short-circuit current of 22.01 mA/cm^2^, and a fill factor of 73.30% (Table 2). Under 1000 h AM 1.5G illumination, the TG-FASnI_3_ cell encapsulated in the N_2_ glove box still maintains more than 95% of its initial efficiency. The use of PEG makes the work function of the hole transport layer adjustable, which can effectively reduce the energy-level mismatch between FASnI_3_ and PEDOT:PSS without damaging the stability of the device, and endows the device with high repeatability and negligible hysteresis, which effectively improves the device performance.

#### 3.4.2. PEDOT:PSS/Poly-TPD 

**Figure 19:**
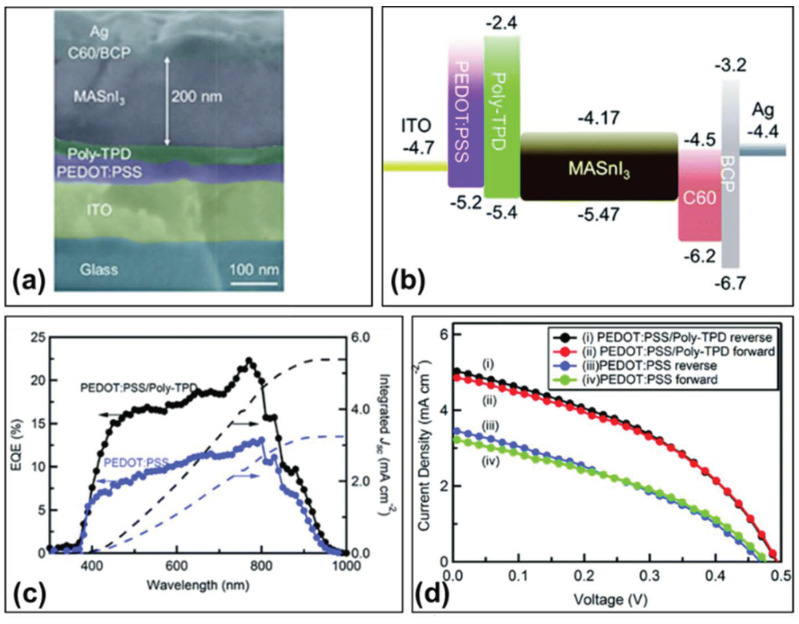
(**a**) Cross-sectional SEM image of the PEDOT:PSS/Poly-TPD-based perovskite solar cell. (**b**) Schematic diagram of the energy band of the device. (**c**) External quantum efficiency and (**d**) Current density–voltage curves of MASnI_3_ perovskite cells using PEDOT:PSS and PEDOT:PSS/Poly-TPD as electron selective layers [91]. Reprinted with permission from Ref. [93]. Copyright 2016 The Royal Society of Chemistry.

Typically, poly[*N*,*N*′-bis(4-butylphenyl)-*N*,*N*′-bis(phenyl)benzidine] (Poly-TPD) was used as the hole transport layer as well as the electron blocking layer proposed in [96], Yu et al. used PEDOT:PSS/Poly-TPD as the hole transport layer and prepared the device ITO/PEDOT:PSS/Poly-TPD/MASnI_3_/C_60_/BCP/Ag (Figure 19a) [91]. The PEDOT:PSS layer was deposited by spin-coating from aqueous solution and the Poly-TPD layer was deposited by spin-coating from 10 mg/mL of chlorobenzene solution. The final solar cell achieved a power conversion efficiency of 1.7%, with an open-circuit voltage of 377 mV, a short-circuit current density of 12.1 mA/cm^2^, and a fill factor of 36.6% (Table 2). The HOMO of MASnI_3_ is −5.47 eV, and the energy level gap between PEDOT:PSS/MASnI_3_ was 0.27 eV; the HOMO of Poly-TPD was −5.40 eV, thus the energy level gap between Poly-TPD/MASnI_3_ was only 0.07 eV (Figure 19b). By combining the Poly-TPD layer with the PEDOT:PSS layer, the external quantum efficiency values were enhanced over the entire wavelength range, indicating that the charge carrier recombination was reduced with the introduction of the Poly-TPD layer (Figure 19c). The perovskite solar cell with PEDOT:PSS/Poly-TPD hole transport bilayer showed a higher short-circuit current density (5.1 mA/cm^2^) and a higher open-circuit voltage (494 mV) than that of devices with only PEDOT:PSS layer (a short-circuit current density of 3.5 mA/cm^2^ and an open-circuit voltage of 470 mV) (Figure 19d). In general, the introduction of Poly-TPD enlarges the grain size of perovskite film deposited on it more than that of perovskite film deposited on PEDOT:PSS, which is conducive to more efficient charge extraction. Moreover, the use of Poly-TPD layer reduces charge recombination and improves the energy level matching effect with perovskite layer. Finally, the open-circuit voltage and short-circuit current of the device are improved.

#### 3.4.3. LiF/PEDOT:PSS

**Figure 20:**
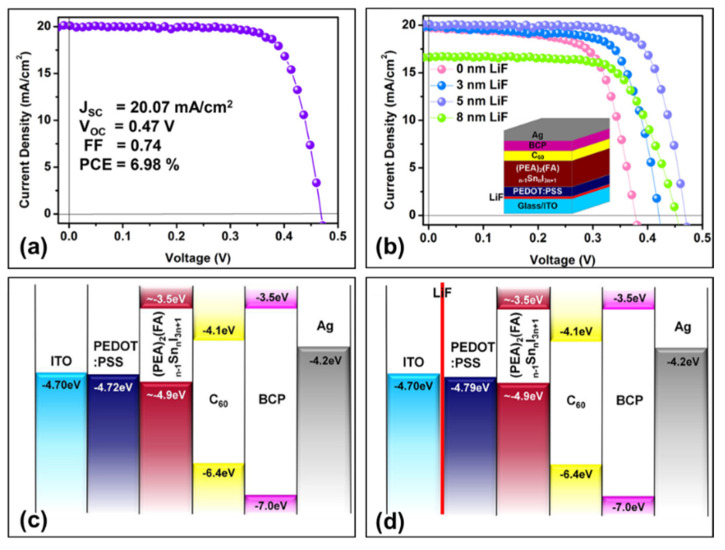
(**a**) The current density–voltage curve of the champion device. (**b**) Current density–voltage curves for devices with different LiF layer thicknesses; the inset shows the device structure of the perovskite solar cell. Energy band alignment diagrams for devices (**c**) without and (**d**) with ultra-thin LiF layers [92]. Reprinted with permission from Ref. [94]. Copyright 2018 American Chemical Society.

Ran et al. inserted LiF between the ITO and PEDOT:PSS layer to promote fast hole extraction, and with (PEA)_2_(FA)_n-1_Sn_n_I_3n+1_ as the perovskite light-absorption layer. They prepared 2D/3D tin-based perovskite solar cells ITO/LiF/PEDOT:PSS/perovskite/C_60_/BCP/Ag (Figure 20b) [92]. The device showed a maximum photoelectric conversion efficiency of 6.98%, a short-circuit current density of 20.07 mA/cm^2^, an open-circuit voltage of 470 mV, and a fill factor of 74% (Figure 20a,b and Table 2). After inserting LiF between ITO and PEDOT:PSS, the tunnelling effect of the ultrathin LiF insulating layer itself can reduce the energy barrier at the ITO/PEDOT:PSS interface, and the higher wettability of the LiF layer can improve the morphology of PEDOT:PSS layer, so the holes collected in PEDOT:PSS can be rapidly extracted by LiF through ITO for carrier extraction, which in turn effectively suppresses carrier recombination at the PEDOT:PSS/ITO interface. A high short-circuit current density and fill factor could be achieved based on this device. On the other hand, due to the different wettability of different thicknesses of LiF layers, it will change the molecular orientation of the PEDOT:PSS films, which finally adjusts the work function and makes the energy level well matched between the hole transport layer and the perovskite light absorber layer. The well-matched energy level not only reduced the charge carrier recombination between the ITO/PEDOT:PSS interface, it also increased the hole extraction, which further improved the open-circuit voltage and fill factor of the device (Figure 20c,d) [97,98,99,100,101]. The LiF insulating layer could (i) lower the work function of PEDOT:PSS, (ii) reduce the energy barrier at the ITO/PEDOT:PSS interface and (iii) reduce the charge recombination at the interface. Due to the improved hole extraction at the ITO/PEDOT:PSS interface, this bifunctional LiF layer further improves the open-circuit voltage and filling factor of the perovskite devices.

The above three methods all optimize the energy level match between hole transport layer and perovskite layer: with the addition of PEG in PEDOT:PSS, the energy level difference between 0.2% PEG-PEDOT:PSS (−4.79 eV) and FASnI_3_ (−4.74 eV) is only 0.05 eV. While the other two methods both add a thin layer on top of the PEDOT:PSS layer, the Poly-TPD energy level (−5.40 eV) matches better with MASnI_3_ (−5.47 eV), and the LiF layer changes the PEDOT:PSS function from −4.72 eV to −4.79 eV, which matches well with the tin-based perovskite layer (−4.90 eV). Furthermore, the addition of a functional layer has the effect of optimizing the device performance, for example, the Poly-TPD layer can play the role of blocking electrons and reducing the charge carrier recombination; the ultra-thin LiF layer can allow the holes to be easily collected in PEDOT:PSS and rapidly extracted to ITO based on its tunnelling effect.

## 4. Inorganic Materials

The hole transport materials used for perovskite solar cells are usually divided into organic and inorganic materials. Organic materials mainly include Spiro-OMeTAD, PTAA, PEDOT:PSS and TPE as mentioned above. Scientists have also replaced organic materials with inorganic materials, which exhibit better stability and higher carrier transport mobility, such as NiOx, CuSCN, etc. These materials have also been used to prepare inverted structure of perovskite solar cell devices.

### 4.1. Inorganic NiOx

**Figure 21:**
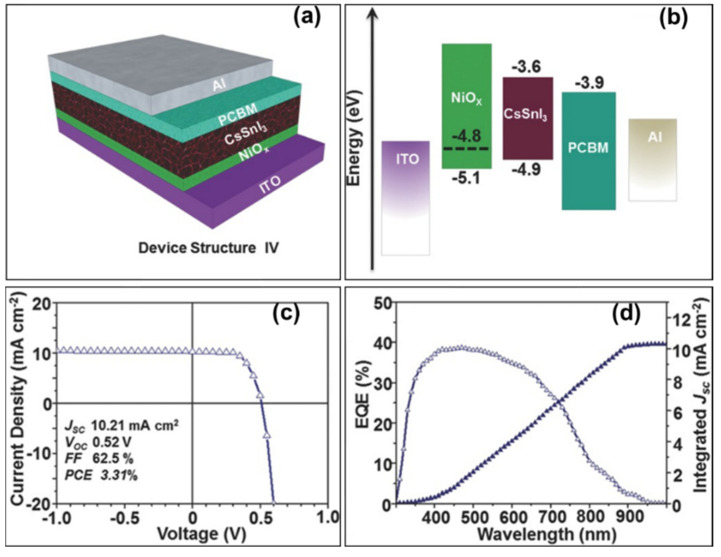
(**a**) Schematic diagram of the CsSnI_3_ device structure using NiOx as the hole transport layer. (**b**) Device energy level diagram (dashed lines indicate NiOx work function). (**c**) Current density–voltage diagram of the CsSnI_3_ device. (**d**) Corresponding external quantum efficiency spectra [40]. Reprinted with permission from Ref. [39]. Copyright 2016 John Wiley and Sons.

With NiOx as the hole transport layer and B-γ-CsSnI_3_ as the perovskite light absorption layer, Wang et al. and Zhou et al. prepared photovoltaic devices with the structure ITO/NiOx/CsSnI_3_/PCBM/Al (Figure 21a) [40]. NiOx dense films were prepared by magnetron sputtering process to deposit 10 nm NiOx films and then spin-coated nickel precursor solution on them. By adjusting the B-γ-CsSnI_3_ grain crystallization and optimizing the device structure, a photovoltaic conversion efficiency of up to 3.31% was obtained without any additives, corresponding to an open-circuit voltage of 520 mV, a short-circuit current density of 10.21 mA/cm^2^ and a fill factor of 62.50% (Figure 21c,d, Table 3). The work function of the prepared NiOx layer is about −4.80 eV (Figure 21b), making the energy level of NiOx layer well-matched with that of the B-γ-CsSnI_3_ perovskite film and favorable for the hole carrier transport.

**Figure 22:**
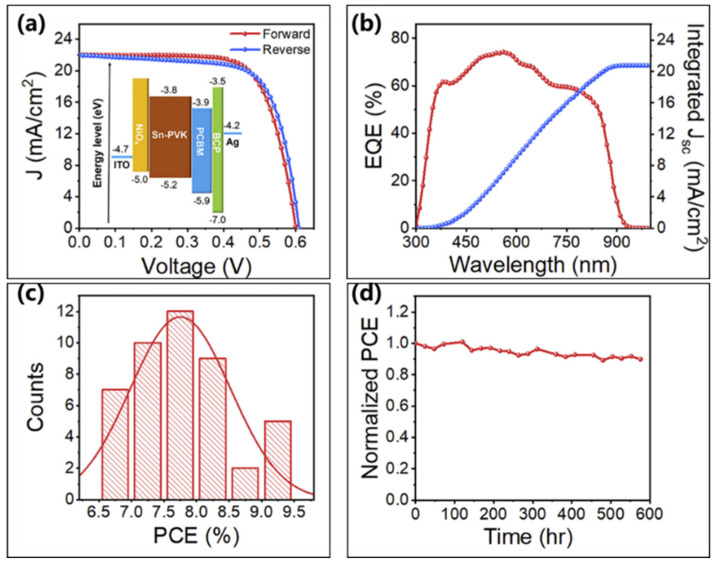
(**a**) The current density–voltage curves for forward and reverse scan mode devices; the inset shows device energy level diagram. (**b**) The external quantum efficiency curve of the device. (**c**) Histogram of the power conversion efficiency of 45 devices. (**d**) Normalized efficiency curve for devices stored in the N_2_ glove box [102]. Reprinted with permission from Ref. [100]. Copyright 2018 Elsevier.

With NiOx as the hole transport layer, and with 2D/3D structured PEA_2_FASn_2_I_7_ perovskite material as the light absorb layer, Wang et al. prepared devices with the structure of ITO/NiOx/Sn-PVSK/PCBM/BCP/Ag [102]. The valence band of 2D/3D perovskite matches well with that of NiOx, and the conduction band bottom is higher than that of [6,6]-phenyl-C61-butyric acid methyl ester, which allows the carriers to efficiently act as transporters. The hole transport layer precursor solution was obtained by dissolving NiOx nanocrystals in a mixed solution (deionized water: isopropanol 3:1, *v*/*v*) and filtering with 0.22 μm polytetrafluoroethylene (PTFE). Finally, the 5% NH_4_SCN-doped perovskite solar cells showed the highest power conversion efficiency of 9.41% under AM 1.5G light illumination, corresponding to a current density of 22 mA/cm^2^, an open-circuit voltage of 610 mV and a fill factor of 70.10% (Figure 22a, Table 3). The short-circuit current density value (20.80 mA/cm^2^) from the external quantum efficiency curve agrees well with the device short-circuit current density (Figure 22b). Moreover, the histogram of the power efficiency of 45 devices showed excellent reproducibility (Figure 22c). The unencapsulated device in N_2_ filled glove box were quite stable for durations up to 600 h (Figure 22d).

In 2021, Wang et al. prepared tin-based perovskite photovoltaic devices with the structure of ITO/NiOx/WSe_2_/FASnI_3_/PCBM/BCP/Ag, using NiOx as the hole transport layer, FASnI_3_ perovskite material as the light-absorbing layer, and WX_2_ flakes as the charge transport interlayer [103]. The device achieved a power conversion efficiency of 10.47%, corresponding to an open-circuit voltage of 630 mV, a short-circuit current density of 22.71 mA/cm^2^, and a fill factor of 73% (Table 3). Compared with NiOx, the WX_2_ layer has high hole mobility and higher valence band level, which makes the hole transport layer NiOx/WSe_2_ and the perovskite film better matched in energy level and the charge carrier transport more efficient. In summary, NiOx is a type of low-cost hole transport material and can be prepared by physical layer deposition, magnetron sputtering and sol-gel processing; notably, it can be prepared at low temperatures, which provides the possibility of flexible tin perovskite devices. At present, the power conversion efficiency of NiOx-based perovskite device is still low. To further improve the device performance, we need to optimize the preparation of NiOx thin films and improve the light transmission and conductivity of NiOx through reasonable doping.

### 4.2. Inorganic CuSCN

**Figure 23:**
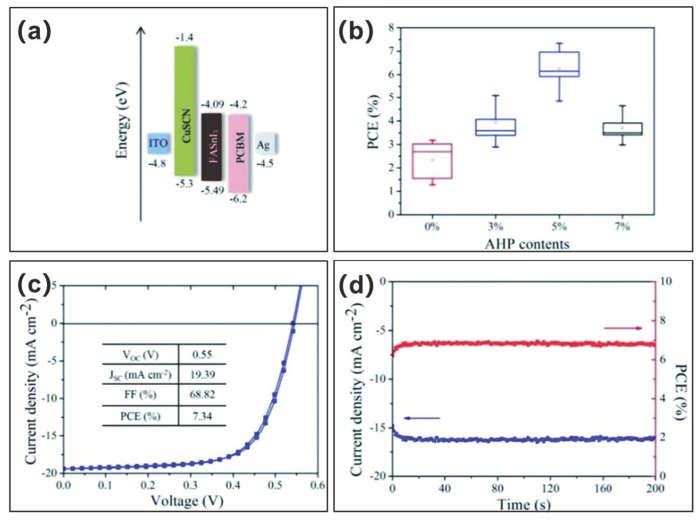
(**a**) Energy level diagram of the devices. (**b**) Distribution of power conversion efficiency of devices prepared with different AHP doping. (**c**) Current density–voltage curves of the champion performance devices. (**d**) Stable power efficiency and photocurrent density of the devices measured at a bias voltage of 430 mV [104]. Reprinted with permission from Ref. [102]. Copyright 2019 The Royal Society of Chemistry.

CuSCN is a type of high hole mobility, high transparent, high stable, and low-cost hole transport layer. Moreover, it has a well-matched energy level with FASnI_3_ perovskite (CuSCN: −5.30 eV; FASnI_3_: −5.02 eV; Figure 23a). With CuSCN as the hole transport layer, and with NH_4_H_2_PO_2_(AHP)-doped FASnI_3_ perovskite as the light absorbing layer, Cao et al. prepared ITO/CuSCN/FASnI_3_/PCBM/Ag structure devices [104]. The CuSCN was dissolved in ammonia (10 mg/mL) and applied via spin-coating on top of the ITO substrate (Figure 23a). The AHP additive introduced in the perovskite solution reduced the oxidation of Sn^2+^ to Sn^4+^, improved the film morphology, reduced the trap density, and optimized the energy matching between the hole transport layer and the perovskite layer. The final champion device (5 mol% AHP) achieved a power conversion efficiency of 7.34%, corresponding to a short-circuit current of 19.39 mA/cm^2^, an open-circuit voltage of 550 mV and a fill factor of 68.8% (Figure 23b,c, Table 3). In addition, these devices exhibited good long-term stability in both pure nitrogen and ambient air (Figure 23d). Notably, the open-circuit voltage reached up to 550 mV, which was one of the highest values for FASnI_3_-based devices. This was attributed to the reduced defect density in the FASnI_3_ films and the effective energy alignment between FASnI_3_ and CuSCN.

## 5. Conclusions

Although the efficiency of tin-based perovskite solar cells has exceeded 14%, there is still an urgent need to improve device efficiency and stability. In this work, we summarized and analyzed the progress of tin-based perovskite solar cells in recent years, especially from the perspective of the hole transport layer. The efficient and stable tin-based perovskite solar cells were obtained via improvement of the components, structure and additives of the hole transport layer. In selecting an appropriate hole transport material, the chosen material requires an excellent ability to extract holes from the perovskite layer as well as its own hole transport ability, which are mainly based on the following two points: the energy levels between the perovskite layer and the hole transport layer should be well matched; the maintenance of proper contact between the hole transport layer and the perovskite interface, which greatly affect the reproducibility and stability of the device. The current high-efficiency tin-based perovskite solar cells commonly utilize PEDOT:PSS as the hole transport material; however, the hydrophilicity and acidity of PEDOT:PSS are damaging to device stability, thus necessitating further optimization of the PEDOT:PSS layer. Finally, the band gap of tin-based perovskite materials can reach the ideal Shockley–Queisser equilibrium limit theory (1.34 eV), and tin is an environmentally heavy metal element. Therefore, the research of tin-based perovskite photovoltaic technology would have an important place in solar cell development, and the attention of researchers to the hole transport layer will continue to increase. 

## Figures and Tables

**Table 1:** Performance of tin-based perovskite solar cells based on Spiro-OMeTAD as hole transport layer.

Perovskite	Device Structure	Voc (mV)	Jsc (mA/cm^2^)	FF (%)	PCE (%)	Ref.
**B-γ-CsSnI_3_**	ITO/TiO_2_/PVSK/Spiro-OMeTAD/Au	480	8.11	19.80	0.77	[40]
**en-FASnI_3_**	FTO/c-TiO_2_/mp-TiO_2_/PVSK/Spiro-OMeTAD/Au	429	13.38	30.79	1.77	[41]
**CsSnI_3_ + 20 mol%SnF_2_**	FTO/c-TiO_2_/mp-TiO_2_/PVSK/Spiro-OMeTAD/Au	240	25.80	30.00	1.87	[42]
**CsSnBr_3_**	FTO/c-TiO_2_/mp-TiO_2_/PVSK/Spiro-OMeTAD + Li-TFSI + tBP/Au	410	9.00	58.00	2.10	[43]
**FASnI_3_**	FTO/TiO_2_+PVSK/Spiro-OMeTAD + Li-TFSI + tBP/Au	238	24.45	36.00	2.10	[44]
**MASnBr_3_**	FTO/c-TiO_2_/PVSK/Spiro-OMeTAD + Li-TFSI + tBP/Au	236	0.03	25.60	0.002	[45]
**MASnI_3_**	FTO/c-TiO_2_/mp-TiO_2_/PVSK/Spiro-OMeTAD + H-TFSI + tBP/Au	880	16.8	42.00	6.40	[25]
**MASnI_3_**	FTO/c-TiO_2_/mp-TiO_2_/PVSK/Spiro-OMeTAD + H-TFSI + 2,6-lutidine/Au	680	16.30	48.00	5.23	[26]
**MASnI_2_Br**	FTO/c-TiO_2_/mp-TiO_2_/PVSK/Spiro-OMeTAD + H-TFSI + 2,6-lutidine/Au	770	14.38	50.00	5.48	[26]
**MASnIBr_2_**	FTO/c-TiO_2_/mp-TiO_2_/PVSK/Spiro-OMeTAD + H-TFSI + 2,6-lutidine/Au	820	12.30	57.00	5.73	[26]
**MASnBr_3_**	FTO/c-TiO_2_/mp-TiO_2_/PVSK/Spiro-OMeTAD + H-TFSI + 2,6-lutidine/Au	880	8.26	59.00	4.27	[26]
**FASnI_3_(25mol% Br)**	FTO/c-TiO_2_/mp-TiO_2_/PVSK/Spiro-OMeTAD + H-TFSI + 2,6-lutidine/Au	414	19.80	66.90	5.50	[46]

**Table 2:** Performance parameters of tin-based perovskite solar cells based on organic materials as hole transport layer.

Perovskite	Device Structure	Voc (mV)	Jsc (mA/cm^2^)	FF (%)	PCE (%)	Ref.
**BA_2_(FA)_n-1_SnnI_3n+1_**	FTO/c-TiO_2_/mp-TiO_2_/BA-PVSK/PTAA + Li-TFSI + tBP/Au	420	23.98	40.21	4.04	[49]
**OA_2_(FA)_n-1_SnnI_3n+1_**	FTO/c-TiO_2_/mp-TiO_2_/OA-PVSK/PTAA + Li-TFSI + tBP/Au	391	20.21	37.84	2.99	[49]
**DA_2_(FA)_n-1_SnnI_3n+1_**	FTO/c-TiO_2_/mp-TiO_2_/DA-PVSK/PTAA + Li-TFSI + tBP/Au	325	16.48	44.95	2.41	[49]
**MASnI_3_**	FTO/c-TiO_2_/mp-TiO_2_/PVSK/PTAA + Li-TFSI + tBP/Au	300	26.10	30.00	1.90	[50]
**MASnI_3_**	FTO/c-TiO_2_/mp-TiO_2_/PVSK/PTAA + Li-TFSI + tBP/Au	230	26.00	39.00	2.30	[51]
**FASnI_3_**	FTO/c-TiO_2_/mp-TiO_2_/PVSK/PTAA + TPFB/Au	380	23.09	60.00	5.27	[52]
**en-FASnI_3_**	FTO/c-TiO_2_/mp-TiO_2_/PVSK/PTAA + TPFB/Au	480	22.54	65.96	7.14	[53]
**MASnI_3_**	FTO/c-TiO_2_/mp-TiO_2_/PVSK/PTAA + TPFB/Au	273	17.80	39.00	1.86	[47]
**(BA)_2_MA_3_Sn_4_I_13_**	FTO/c-TiO_2_/mp-TiO_2_/infiltrated PVSK/PVSK capping layer/PTAA + TPFB/Au	229	24.10	45.70	2.53	[54]
**en-MASnI_3_**	FTO/c-TiO_2_/mp-TiO_2_/PVSK/PTAA + TPFB/Au	428	24.28	63.72	6.63	[55]
**MASnI_3_**	FTO/c-TiO_2_/mp-TiO_2_/PVSK/PVSK/PTAA + TPFB/Au	378	19.92	51.73	3.89	[56]
**FASnI_3_(10%PN)**	FTO/c-TiO_2_/mp-TiO_2_/PVSK/PTAA + TPFB/Au	435	22.15	60.67	5.85	[57]
**en-FASnI_3_**	FTO/SnO_2_-CPTA/PVSK/PTAA + TPFB/Au	720	16.45	65.00	7.40	[58]
**MASnBr_3_**	FTO/c-TiO_2_/PVSK/P3HT/Au	498	4.27	49.10	1.12	[45]
**Cs_2_SnI_6_**	FTO/c-ZnO/n-ZnO/PVSK/P3HT/Ag	520	3.20	51.50	1.00	[59]
**Cs_2_SnI_6_**	FTO/TiO_2_/PVSK/P3HT/Ag	510	5.41	35.00	1.00	[60]
**en-FASnI_3_**	FTO/c-TiO_2_/mp-TiO_2_/en-FASnI_3_/TPE/Au	453	22.60	67.00	6.85	[41]
**FASnI_3_(10mol%SnF_2_)**	ITO/PEDOT:PSS/PVSK/C60/BCP/Ag	465	22.07	60.00	6.22	[61]
**MASnI_3_(LT-M** **ix)**	ITO/PEDOT:PSS/MASnI_3_/C60/BCP/Ag	450	11.82	40.00	2.14	[62]
**PP-FASnI_3_**	ITO/PEDOT:PSS/PP-FASnI_3_/C60/BCP/Ag	330	17.78	67.9	3.98	[63]
**(FA)_0.75_(MA)_0.25_SnI_3_**	ITO/PEDOT:PSS/PVSK/C60/BCP/Ag	610	21.20	62.70	8.12	[64]
**FASnI_3_**	ITO/PEDOT:PSS/FASnI_3_/C60/BCP/Cu	450	24.87	63.00	7.05	[65]
**FASnI_3_:PMMA**	ITO/PEDOT:PSS/FASnI_3_:PMMA/PCBM/Ag	482	13.17	57.00	3.62	[66]
**FASnI_3_(2.5%N_2_H_5_Cl)**	ITO/PEDOT:PSS/FASnI_3_/PCBM/BCP/Ag	455	17.63	67.30	5.40	[67]
**(PEAI)_0.1_FA_0.9_SnI_3_(5%FASCN)**	ITO/PEDOT:PSS/PVSK/PCBM/Al	530	21.80	66.50	7.66	[68]
**(PEAI)_0.08_FA_0.92_SnI_3_**	ITO/PEDOT:PSS/PVSK/C60/BCP/Al	525	24.10	71.00	9.00	[69]
**FASnI_3_ + SnF_2_ + TMA**	ITO/PEDOT:PSS/FASnI_3_/C60:1 wt% TBAI/Ag	470	22.45	67.80	7.09	[70]
**GA_x_FA_(0.98−x)_SnI_3_–1% EDAI_2_**	ITO/PEDOT:PSS/PVSK/C60/BCP/Ag	619	21.20	72.90	9.60	[71]
**FA_0.98_EDA_0.01_SnI_3_(0.05 mM DAE)**	FTO/PEDOT:PSS/PVSK/C60/BCP/Ag/Au	600	23.09	73.00	10.18	[72]
**FASnI_3_(3%5-AVAI)**	ITO/PEDOT:PSS/FASnI_3_/PCBM/BCP/Ag	592	18.89	62.30	7.00	[73]
**FASnI_3_(PEABr)**	ITO/PEDOT:PSS/PVSK/PCBM/BCP/Al/Ag	540	22.64	64.00	7.86	[74]
**EA_0.1_(FA_0.75_MA_0.25_)_0.9_SnI_3_**	ITO/PEDOT:PSS/PVSK/PCBM/C60/BCP/Ag/Au	470	17.45	66.00	5.41	[75]
**(BA_0.5_PEA_0.5_)_2_FA_3_Sn_4_I_13_**	ITO/PEDOT:PSS/PVSK/C60/LiF/Al	600	21.82	66.73	8.82	[76]
**FASnI_3_(15%PPAI)**	ITO/PEDOT:PSS/PVSK/C60/BCP/Ag	560	23.22	72.60	9.44	[77]
**EA_0.08_-FASnI_3_/PEA_2_FASn_2_I_7_**	ITO/PEDOT:PSS/PVSK/C60/BCP/Al	510	23.75	70.00	8.40	[78]
**AVA_2_FA_n−1_Sn_n_I_3n+1_(10%NH_4_Cl)**	ITO/PEDOT:PSS/PVSK/PCBM/BCP/Ag	610	21.00	68.00	8.71	[79]
**PEA_x_FA_1−x_SnI_3_(NH_4_SCN)**	ITO/PEDOT:PSS/PVSK/ICBA/BCP/Ag	940	17.40	75.00	12.40	[80]
**FA_0.9_PEA_0.1_SnI_3_**	ITO/PEDOT:PSS/PVSK/ICBA BCP/Al	651	16.88	64.00	7.05	[81]
**FASnI_3_-50%LFA**	ITO/PEDOT:PSS/PVSK/C60/BCP/Ag	616	22.08	72.80	9.90	[82]
**(FA_0.9_EA_0.1_)_0.98_EDA_0.01_SnI_3_(GeI_2_)**	FTO/PEDOT:PSS/PVSK/C60/BCP/Ag/Au	840	20.38	74.00	13.24	[83]
**FASnI_3_(5% PHCl)**	ITO/PEDOT:PSS/FASnI_3_/C60/BCP/Ag	760	23.50	64.00	11.40	[84]
**FA_0.8_GA_0.2_SnI_3_/AN_2_FA_n−1_Sn_n_I_3n+1_**	ITO/PEDOT:PSS/PVSK/C60/BCP/Ag	645	21.10	76.30	10.40	[85]
**CsSnI_3_**	ITO/PEDOT:PSS/CsSnI_3_/C60/BCP/Cu	630	19.70	66.10	8.20	[86]
**FASnI_X_Br_3-X_(5% PhNHNH_3_Cl)**	ITO/PEDOT:PSS/PVSK/C60/BCP/Ag	760	22.95	70.98	12.38	[87]
**FA_0.9_SnI_3_(10% FPEABr)**	ITO/PEDOT:PSS/PVSK/ICBA/BCP/Al	828	24.50	69.40	14.03	[88]
**FASnI_3_**	FTO/(PEG-)PEDOT:PSS/PVSK/PCBM/BCP/Ag	370	22.06	62.70	5.12	[89]
**TG-FASnI_3_**	ITO/PEG-PEDOT:PSS/PVSK/C60+BCP/Ag	695	22.01	73.30	11.22	[90]
**MASnI_3_**	ITO/PEDOT:PSS/Poly-TPD/PVSK/C60/BCP/Ag	377	12.10	36.60	1.70	[91]
**(PEA)_2_(FA)_n-1_Sn_n_I_3n+1_**	ITO/LiF/PEDOT:PSS/PVSK/C60/BCP/Ag	470	20.07	74.00	6.98	[92]

**Table 3:** Performance parameters of tin-based perovskite solar cells based on NiOx and CuSCN as hole transport layer.

Perovskite	Device Structure	Voc (mV)	Jsc (mA/cm^2^)	FF (%)	PCE (%)	Ref.
**B-γ-CsSnI_3_**	ITO/NiOx/PVSK/PCBM/Al	520	10.21	62.50	3.31	[40]
**2D(PEA_2_FASn_2_I_7_)/3D(FASnI_3_)**	ITO/NiOx/PVSK/PCBM/BCP/Ag	610	22.00	70.10	9.41	[102]
**FASnI_3_**	ITO/NiOx/WSe_2_/PVSK/PCBM/BCP/Ag	630	22.71	73.20	10.47	[103]
**FASnI_3_(5 mol% AHP)**	ITO/CuSCN/FASnI_3_/PCBM/Ag	550	19.39	68.80	7.34	[104]

## Data Availability

Data availability is not applicable to this article as no new data were created or analyzed in this study.
